# Prevalence of *Brucella* in dogs in China: a systematic review and meta-analysis—Epidemiological analysis of canine brucellosis

**DOI:** 10.3389/fvets.2024.1515405

**Published:** 2025-02-13

**Authors:** Mei-Mei Xiang, Hong-Yun Jiang, Qiu-chi Jiang, Yi-fan Zhang, Jia-yu Yu, Lian-Min Li, Qi Wang, Ting Li, Wen-tao Xiang, Chong-bin Chen, Zheng-yang Xie, Xue Leng, Qian-zhen Zhang, Fei Liu, Jian-Ming Li

**Affiliations:** ^1^College of Animal Science and Technology, Jilin Agricultural University, Changchun, Jilin, China; ^2^Laboratory of Production and Product Application of Sika Deer of Jilin, Jilin Agricultural University, Changchun, Jilin, China; ^3^Key Lab of Animal Production, Product Quality and Security, Ministry of Education, Jilin Agricultural University, Jilin, China; ^4^Jilin Beiyao Traditional Chinese Medicine Pharmaceutical Group Co., Changchun, Jilin, China; ^5^College of Chinese Medicine Materials, Jilin Agricultural University, Changchun, Jilin, China

**Keywords:** *Brucella*, meta-analysis, dogs, China, Brucellosis

## Abstract

**Introduction:**

Brucellosis is a zoonotic disease that affects both dogs and humans. With the increase in dog ownership, the risk of transmission has risen for both adults and children.

**Methods:**

This study used meta-analysis to comprehensively analyze the prevalence of canine brucellosis in China and to identify the relevant factors affecting its transmission.

**Result:**

We conducted a meta-analysis of 38 studies published between January 1983 and March 2024, sourced from six databases. The results showed a higher prevalence of canine brucellosis in northern and northwestern regions of China, with Xinjiang having the highest prevalence (19.77%) and Hunan the lowest (0.23%). Significant differences were found in positivity rates across different diagnostic methods (*P* < 0.05), with ELISA yielding the highest positivity rate (11.6%) and PCR and SAT the lowest (3.3%). The positivity rate of stray dogs (22.6%) was significantly higher than that of other dog sources (*P* < 0.05). Furthermore, environmental factors, such as temperature and altitude, were identified as influencing the incidence of brucellosis.

**Discussion:**

In conclusion, canine brucellosis is prevalent across China, with detection methods, dog sources, and environmental factors contributing to the varying incidence rates. We recommend regular brucellosis testing for pet dogs, improved kennel hygiene, and reduced contact with potentially infected animals.

**Systematic review registration:**

https://www.frontiersin.org/journals/veterinary-science

## 1 Introduction

Brucellosis is a bacterial zoonotic disease that poses a serious threat to both human and animal health. The Ministry of Agriculture and Rural Affairs of China has listed it as a second-class animal disease, and it has been classified by the World Organization for Animal Health (OIE) as a notifiable animal disease (1). *Brucella* mainly includes *Brucella abortus* (*B. abortus*), *Brucella melitensis* (*B. melitensis*), *Brucella suis* (*B. suis*), *Brucella inopinata* (*B. inopinata*), *Brucella canis* (*B. canis*), *Brucella ovis* (*B. ovis*), *Brucella ceti, Brucella pinnipedialis* (*B. pinnipedialis*)*, Brucella rusensis* (*B. rusensis*)*, Brucella microti* (*B. microti*) and *Brucella neotomae* (*B. microti*) (2). Different species of animals (including wild animals and marine animals) are susceptible to brucellosis, especially common cattle, sheep, and pigs. Healthy animals become infected with brucellosis mainly through direct contact or indirect contact with infected people or other animals (3). Clinical manifestations include prolonged fever, hyperhidrosis, arthralgia, hepatosplenomegaly, and can lead to reproductive system disorders in both humans and animals (4). Although brucellosis is rarely fatal, it can lead to complications like infertility and arthritis, making it a significant public health concern. Because *Brucella* is an intracellular pathogen, it is difficult for sensitive antibiotics to enter the cell to exert a therapeutic effect (5). Consequently, once an infection occurs, it can be difficult to cure and can result in serious long-term health consequences. The prevention and control of brucellosis primarily rely on vaccination of animals and their monitoring.

With the growing relationship between dogs and humans, the risk of *Brucella* being transmitted from dogs to humans and domestic animals has gradually increased. *Brucella canis* (*B. canis*) was first isolated in the United States in 1966, and China was the first to isolate *B. canis* from Beagle dogs imported from the United States and Canis lupus families in 1984 (6). Canine brucellosis is mainly caused by the *Brucella canis*, and other species of *Brucella* can also infect dogs. *Brucella canis* is a rough strain (7), which can cause miscarriage in female dogs, epididymitis in male dogs, etc. The clinical symptoms and necropsy changes of this disease often lack obvious characteristics, and the diagnosis requires laboratory examination. Studies have demonstrated that the overall prevalence of canine brucellosis in the pastoral areas of Urumqi, Xinjiang, can reach up to 41.5% (8). Moreover, the positive rate of antibodies in dogs has been reported to be ranging from 8.61% to 42.65% in the provinces of Yunnan, Guizhou, and Sichuan (9). In a follow-up study of 100 patients with acute brucellosis, it was found that the majority had a history of contact with animals such as dogs, sheep, pigs, and cattle, with the highest percentage of exposure being to dogs (79.59%) (10). Additionally, research indicates that pediatric brucellosis cases often involve a history of canine exposure. This suggests that contact with dogs may be a significant risk factor for brucellosis infection (11).

To our knowledge, no systematic review or meta-analysis has been conducted to assess the prevalence of canine brucellosis in China. Comparing prevalence across regions is essential for understanding geographic variations, guiding control strategies, optimizing resources, and raising awareness. Regular studies can help implement targeted preventive measures to control the spread of the disease and protect public health. This study aims to address these gaps by providing a comprehensive meta-analysis of the incidence rates, diagnostic methods, and influencing factors associated with canine brucellosis across different regions and age groups in China.

## 2 Methods

### 2.1 Screening criteria

The article was based on the systematic review and meta-analysis (PRISMA) (12) guidelines for analysis (Supplementary Table S1). We performed a comprehensive search across six databases for all publications concerning canine brucellosis: PubMed, SpringerLink, ScienceDirect, China National Knowledge Infrastructure (CNKI), Wan fang, and VIP Chinese Journal Databases (VIP). The search strategy is detailed in the Supplementary Appendix S1.

We identified and screened articles according to the following Principles:

- The study must specifically involve dogs as the subject of investigation.
- The research must focus on the prevalence of brucellosis in dogs within China.
- The article must clearly specify the total number of dogs sampled as well as the number of dogs testing positive for brucellosis.

Articles that fulfill any of the following Principle will be excluded:

- Other pathogens
- Other species
- Error in data
- Data duplication
- No data
- Overview
- Unable to download.

### 2.2 Data extractions and evaluation system

We filter the following data from the articles that meet the standards, including: region, publication year, sampling years, province, detection method, *Brucella* type, breeding mode, gender, age, quality level, total number of examined dogs and number of *Brucella*-positive dogs.

Extraction of data from articles based on geographical area and geographical factors, including: longitude, latitude, altitude, precipitation, humidity, temperature (maximum and minimum).

We evaluated the quality of the available studies using the Grading of Recommendations Assessment, Development, and Evaluation (GRADE) method (13) (Supplementary Table S3). A scoring system was applied to rate each study. One point was awarded for meeting each of the following criteria: elaboration of random sampling, detection method used, sampling techniques, sampling time, and inclusion of more than four variables in the study. This allowed a maximum score of five points per study. Studies scoring 4 or 5 points were categorized as high quality, those with 2 or 3 points were considered moderate quality, and those with 0–1 points were classified as low quality. Importantly, studies with lower scores were still included in the meta-analysis if they met the inclusion criteria, even if they lacked sufficient detail for in-depth analysis.

### 2.3 Statistical analysis

Meta analysis was performed using the “meta” package (version 4.12-0) in R (version 4.0.0) (14) (Supplementary Table S2). According to the basis of previous studies, double-arcsine transformation (PFT) contrast rate analysis was used for conversion before meta-analysis (15–17) (Table 1). We used a random effect model to combined with subgroup analysis to avoid the heterogeneity caused by paired analysis. *I*^2^ and Cochrane *Q* statistics were used to predict heterogeneity (represented by χ^2^ and *P*-values), typically *I*^2^ value of 25% corresponds to low heterogeneity, 50% to moderate heterogeneity and 75% to high heterogeneity.

**Table 1:** Normal distribution test for the normal rate and the different conversion of the normal rate.

**Conversion form**	***W***	***P***
PRAW	0.70388	1.904e-07
PLN	NaN	NA
PLOGIT	NaN	NA
PAS	0.89069	0.001406
PFT	0.87972	0.0007156

PRAW, original rate; PLN, logarithmic conversion; PLOGIT, logit transformation; PAS, arcsine transformation; PFT, double-arcsine transformation; NaN, meaningless number; NA, missing data.

Publication bias was assessed with funnel plots and Egger's tests (Supplementary Table S4), and adjusted with trim-and-fill analyses. The stability of the results was verified by sensitivity analysis.

We performed subgroup analyses and univariate regression analyses to identify factors contributing to heterogeneity. The factors include quality level (high and others), region (Central China and other regions), sampling year (2010 or before and later of 2010), detection method (ELISA and others), *Brucella* type (R type and S type), gender (female and male), and age (< 1 year and others). In addition, we further assessed the geographic factors, including latitude (20–30° and others), longitude (80–100° and others), altitude (0–1,000 m and others), Rainfall (mm) (0–500 mm and others), Humidity (35–60 and others), mean annual temperature (−5 to 5°C and others).

## 3 Results

### 3.1 Article filtering results

**Figure 1:**
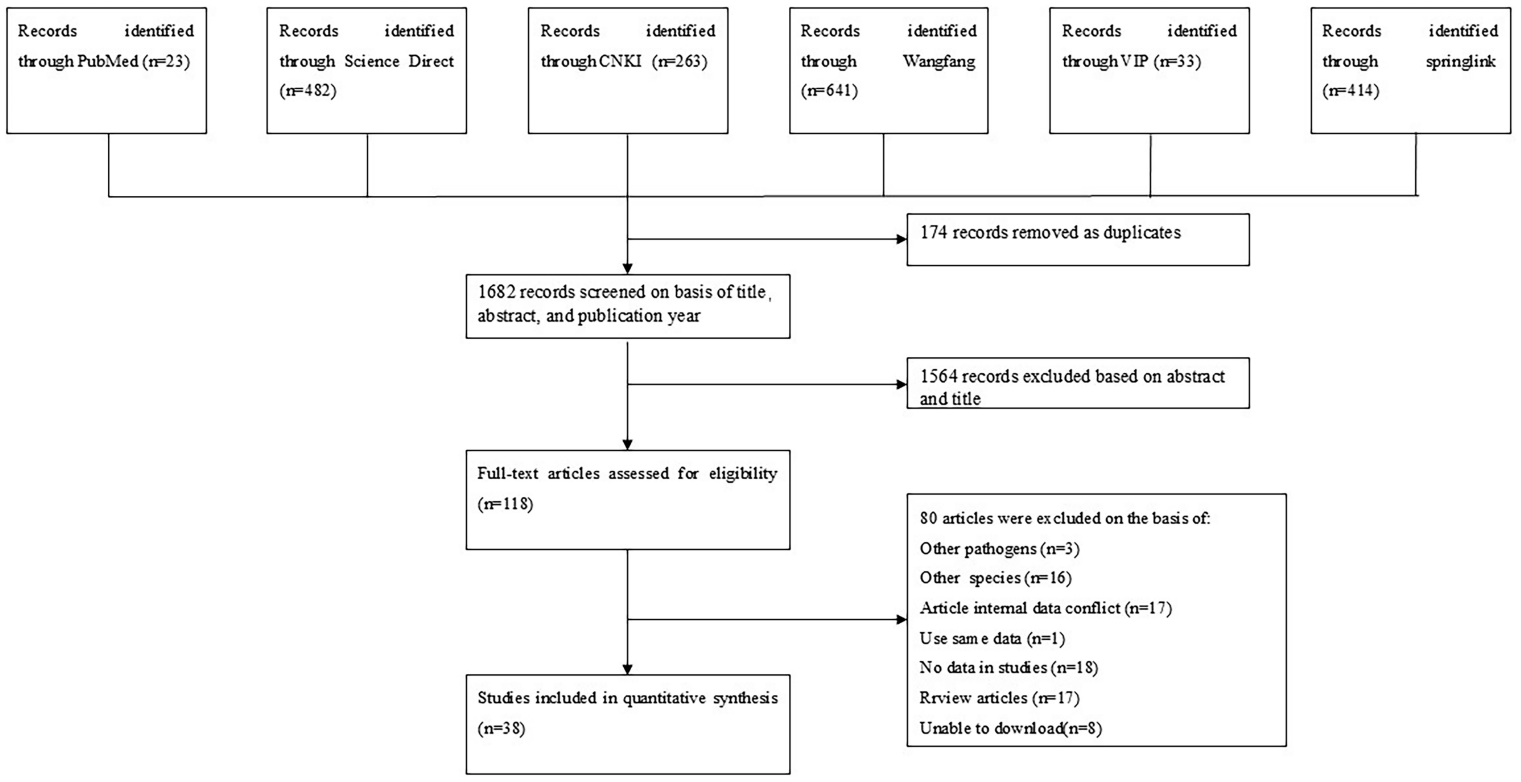
Flow diagram of eligible studies for searching and selecting.

We conducted a search of 1,856 studies across six databases and, based on predefined inclusion and exclusion criteria, selected 38 articles published between January 1983 and March 2024 for analysis (Figure 1). The quality of these articles was assessed according to several criteria, including sampling time, random sampling, detailed sampling methods, and clarity of the detection method. As a result, eight papers were rated 4–5 points, 29 papers were rated 2–3 points, and one paper was rated 0–1 points (Table 2).

**Table 2:** Studies included in the analysis.

**References**	**Sampling time**	**Province**	Detection methods^*^	**No. tested**	**No. positive**	**Quality level^⋆^**
**Central China**						
Yuan (18)	2013–2014	Hunan	ELISA	870	6	High
Lu et al. (19)	2016	Hunan	ELISA	184	0	Middle
**East China**						
Liu et al. (20)	UN	Shanghai	SAT	123	9	Middle
Li et al. (21)	UN	Shanghai	SAT	443	0	Low
Yan et al. (22)	2011–2013	Jiangsu	SAT	348	4	Middle
Tao et al. (23)	UN	Shanghai	SAT	114	12	Middle
Hang et al. (5)	2016–2017	Shandong	ELISA	502	54	Middle
**North China**						
He et al. (24)	2006–2007	Beijing	SAT	415	1	High
Xiang et al. (25)	2008–2009	Beijing	SAT	1,200	21	Middle
Qi et al. (4)	2011–2011	Beijing	SAT	504	7	High
Gao (26)	2009–2010	Inner Mongolia	SAT	196	75	Middle
Wang et al. (27)	2012–2013	Beijing	SAT	38	18	Middle
Xu et al. (28)	UN	Inner Mongolia	SAT	145	2	Middle
Wang et al. (29)	2016–2016	Beijing	PCR	275	0	High
**Northeast China**						
Sun (3)	2018–2019	Liaoning	PCR	201	11	High
**Northwest China**						
Hu et al. (30)	UN	Gansu	SAT	305	3	Middle
Xue and Lu (31)	2007–2009	Gansu	SAT	150	21	High
Lu et al. (32)	2011–2013	Gansu	SAT	1,094	1	Middle
Bao (33)	2014	Qinghai	RBPT	120	1	High
Che et al. (34)	UN	Gansu	SAT	252	11	Middle
Hasibat (35)	2015–2015	Xinjiang	RBPT	381	113	High
Ye et al. (36)	2013–2014	Xinjiang	ELISA	216	39	Middle
Liu et al. (37)	UN	Xinjiang	ELISA	121	15	Middle
Cao et al. (38)	2013–2015	Gansu	SAT	698	1	Middle
Wang et al. (8)	2016	Xinjiang	ELISA	354	90	Middle
Liao et al. (39)	UN	Xinjiang	Test strip	368	52	Middle
**South China**						
Zhang et al. (40)	2006–2007	Guangdong	SAT	1,145	24	Middle
Liang (41)	UN	Guangdong	SAT	442	15	Middle
Chen et al. (42)	UN	Guangdong	SAT	267	35	Middle
Deng (43)	2011–2012	Guangdong	SAT	545	6	Middle
Chen et al. (44)	2012–2016	Guangdong	SAT	4,508	4	Middle
**Southwest China**						
Wang et al. (45)	2003–2009	Guizhou	SAT	315	12	Middle
Wang (46)	2011–2012	Guizhou	SAT	55	3	High
Zhang et al. (47)	UN	Yunnan	PCR	112	11	Middle
Yang et al. (48)	2012–2014	Sichuan	SAT	110	1	Middle
Zhang et al. (49)	2011–2015	Sichuan	SAT	860	13	Middle
Xu et al. (50)	2017–2018	Yunnan	SAT	208	3	High
**UN**						
Di et al. (6)	2007–2009	UN	SAT	4,750	60	Middle

UN^⋆^, unclear.

Detection methods^⋆^: RBPT, Rose Bengal Test; SAT, Serum Agglutination Test; ELISA, Enzyme Linked Immunosorbent Assay.

Quality level^⋆^: High (4–5), Middle (2–3), Low (0–1).

### 3.2 Regional and provincial risk factor assessment results

**Figure 2:**
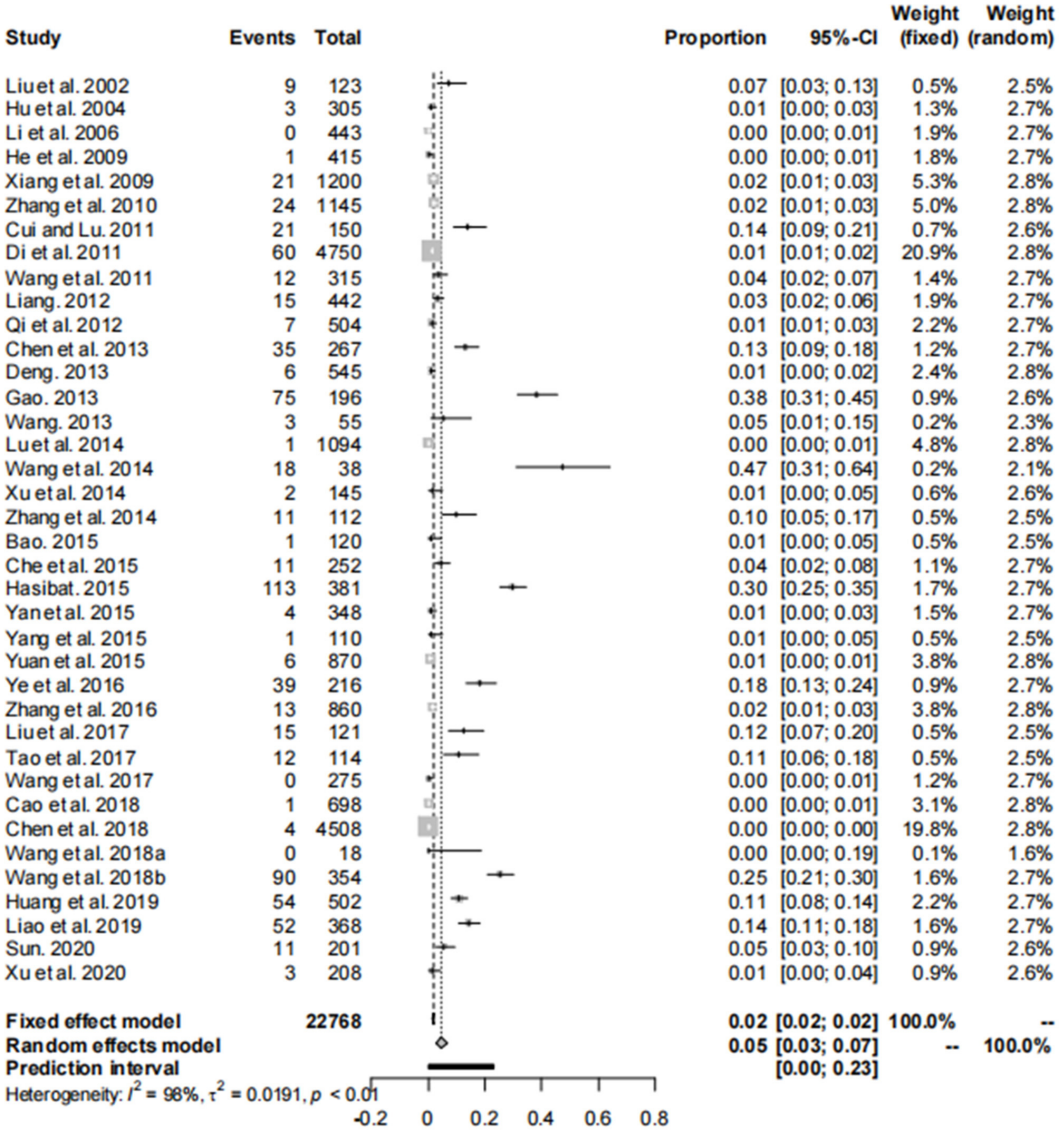
Forest plot of prevalence of the *Brucella* in dogs amongst studies conducted in China.

**Figure 3:**
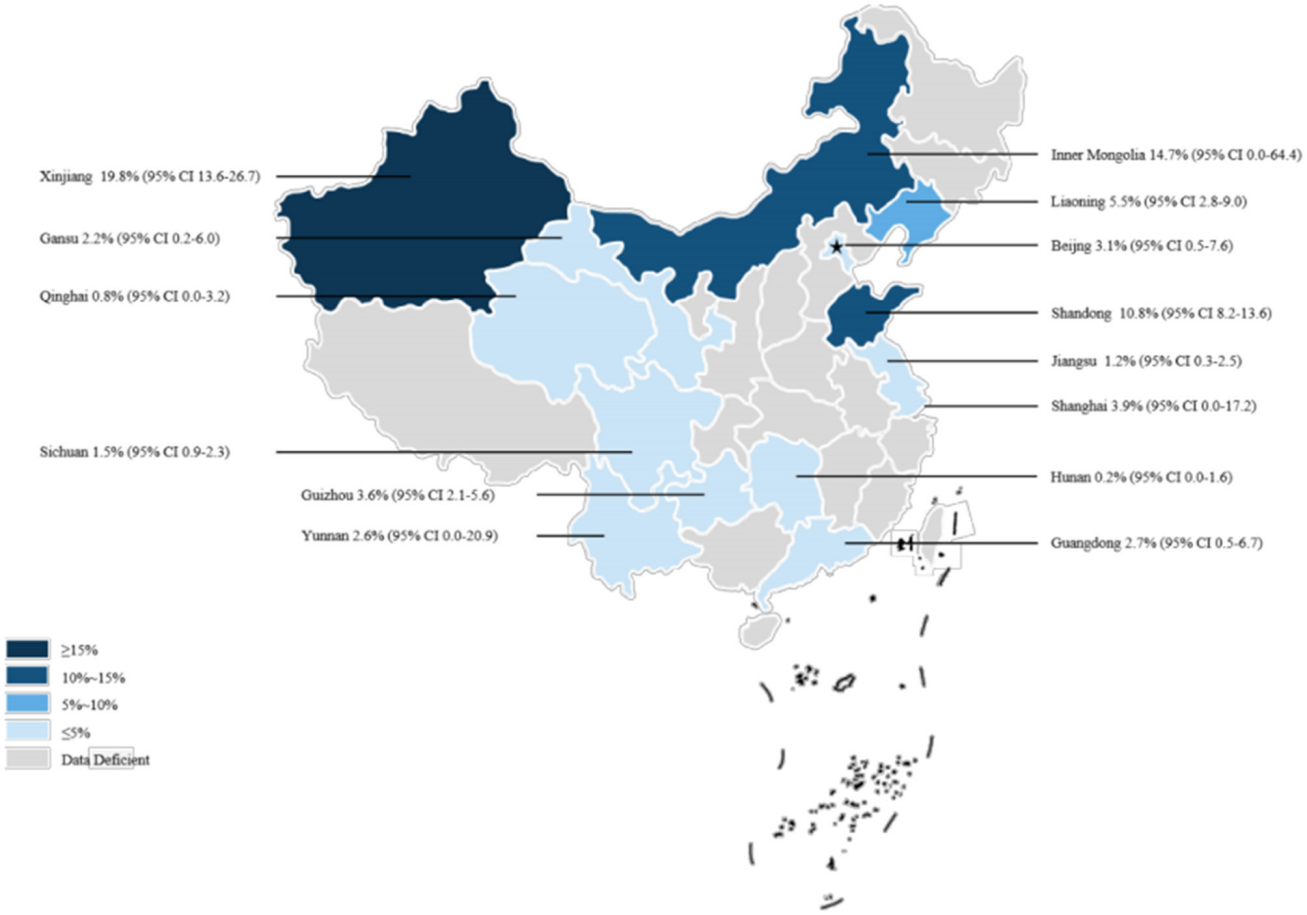
Map of *Brucella* in dogs amongst studies conducted in China.

Our analysis of data from 22,768 dogs across 14 provinces in seven regions of China, as reported in these 38 articles, revealed a canine brucellosis positivity rate of 4.7% (95% Cl: 3.0–6.8, Figure 2, Table 3, Supplementary material S6, S7). Geographically, the highest prevalence of canine brucellosis was found in Northwestern China, at 7.9% (2.3%−16.6%, Table 3, Supplementary material S6, S7); The lowest prevalence of canine brucellosis in Central China at 0.2% (0.0%−1.6%, Table 3, Supplementary material S6, S7). The prevalence of canine brucellosis varies between provinces, with the highest prevalence of canine brucellosis in Xinjiang (19.8%, 95% CI: 13.6%−26.7%, Figure 3, Table 4, Supplementary material S6, S7), followed by Inner Mongolia (14.7%, 95% CI: 0.0%−64.4%, Figure 3, Table 4, Supplementary material S6, S7), Shandong (10.8%, 95% CI: 8.2%−13.6%, Figure 3, Table 4, Supplementary material S6, S7) and Liaoning (5.5%, 95% CI: 2.8%−9.0%, Figure 3, Table 4, Supplementary material S6, S7), brucellosis infection rate was lowest in Hunan (0.2%, 95% CI: 0.0%−1.6%, Figure 3, Table 4, Supplementary material S6, S7).

**Table 3:** Aggregate prevalence of brucellosis in dog in China.

		**No. studies**	**No. tested**	**No. positive**	**% (95% CI)**	**Heterogeneity**			**Univariate meta-regression**	
						χ^2^	***P*** **-value**	*I*^2^ **(%)**	***P*** **-value**	**Coefficient (95% CI)**
Region	Central China	2	1,054	6	0.2% (0.0–16.4)	4.20	0.04	76.2%		
	East China	5	1,530	79	4.2% (0.4–12.1)	127.12	< 0.01	96.9%		
	North China	7	2,773	124	6.3% (1.1–15.1)	311.64	< 0.01	98.1%		
	Northeast China	1	201	11	5.5% (2.8–9.0)	0.00	–	–		
	Northwest China	11	4,059	347	7.9% (2.3–16.6)	710.91	< 0.01	98.6%	0.068	0.100 (−0.007 to 0.208)
	South China	5	6,907	84	2.7% (0.5–6.7)	175.95	< 0.01	97.7%		
	Southwest China	8	1,660	43	2.5% (1.0–4.6)	26.41	< 0.01	73.5%		
Sampling years	2010 or before	19	8,416	214	4.2% (1.5–8.2)	303.77	< 0.01	97.7%	0.603	0.031 (−0.086 to 0.149)
	2010 later	8	11,472	281	3.1% (1.2–6.0)	860.65	< 0.01	97.9%		
Detection method	ELISA	5	2,063	204	11.6% (2.8–25.5)	252.78	< 0.01	98.4%		
	PCR	3	588	22	3.3% (0.0–13.5)	43.30	< 0.01	95.4%		
	RBPT	2	501	114	10.8% (0.0–52.5)	85.69	< 0.01	98.8%		
	SAT	26	19,230	362	3.3% (2.0–4.9)	686.36	< 0.01	96.4%	0.008	−0.121 (−0.210 to −0.032)
	Test strip	1	368	52	14.1% (10.8–17.9)	0.00	–	–		
Type	R	14	9,924	225	3.1% (1.4–5.5)	349.88	< 0.01	96.3%	0.550	0.026 (−0.060 to 0.113)
	S	10	8,879	138	2.3% (0.8–4.5)	230.78	< 0.01	96.1%		
Gender	Female	8	1,274	34	1.7% (1.1–2.5)	56.40	< 0.01	87.6%		
	Male	8	1,595	47	2.2% (1.6–3.0)	56.74	< 0.01	87.7%	0.683	0.025 (−0.094 to 0.143)
Age	< 1 year	7	760	11	0.6% (0.0–2.2)	18.00	< 0.01	66.7%		
	≥1 < 3 year	8	1,221	18	1.1% (0.4–2.1)	13.29	0.07	47.3%		
	≥3 years	9	1,000	28	2.2% (1.0–4.0)	19.51	0.01	59.0%	0.073	0.054 (−0.005 to 0.114)
Category	Breed dog	13	2,603	74	5.1% (2.0–9.4)	178.20	< 0.01	93.3%		
	Domestic dog	21	8,764	392	9.5% (5.4–14.6)	878.65	< 0.01	97.7%		
	Free-range dog	2	1,534	15	0.8% (0.1–2.0)	4.14	0.04	75.9%		
	Outpatient dog	10	2,826	126	5.2% (2.0–10.0)	184.16	< 0.01	95.1%		
	Stray dog	7	369	84	22.6% (18.3–27.2)	6.33	0.39	5.3%	0.0003	0.244 (0.111 to 0.377)
Quality	High	1	443	0	0.0% (0.0–0.2)	0.00	–	–		
	Middle	29	19,616	592	5.2% (3.2–7.6)	1,196.10	< 0.01	97.7%	0.338	0.053 (−0.055 to 0.161)
	Low	8	2,709	162	4.0% (0.4–11.1)	348.96	< 0.01	98.0%		
Total		38	22,768	754	4.7% (3.0–6.8)					

CI, confidence interval; R^2^, proportion of between-study variance explained by joint test with provinces as a covariate.

Detection method: RBPT, Rose Bengal Test; SAT, Serum Agglutination Test; ELISA, Enzyme Linked Immunosorbent Assay.

Type: Brucella colony typing.

Quality level: High (4–5), Middle (2–3), Low (0–1).

**Table 4:** Estimation of the prevalence of brucellosis in dog in the provinces.

**Province**	**No. studies**	**Region**	**No. tested**	**No. positive**	**% Prevalence**	**% (95% CI)**
Hunan	2	Central China	1,054	6	0.23%	0.0–1.6
Jiangsu	1	East China	348	4	1.15%	0.3–2.5
Shandong	1	East China	502	54	10.76%	8.2–13.6
Shanghai	3	East China	680	21	3.90%	0.0–17.2
Inner Mongolia	2	North China	341	77	14.65%	0.0–64.4
Beijing	5	North China	2,432	47	3.06%	0.5–7.6
Liaoning	1	Northeast China	201	11	5.47%	2.8–9.0
Qinghai	1	Northwest China	120	1	0.83%	0.0–3.2
Xinjiang	5	Northwest China	1,440	309	19.77%	13.6–26.7
Gansu	5	Northwest China	2,499	37	2.15%	0.2–6.0
Guangdong	5	South China	6,907	84	2.68%	0.5–6.7
Yunnan	2	Southwest China	172	11	2.61%	0.0–20.9
Guizhou	3	Southwest China	433	16	3.61%	2.1–5.6
Sichuan	3	Southwest China	1,055	16	1.50%	0.9–2.3

### 3.3 Sampling year risk factor assessment results

In 2010 and before, the prevalence of canine brucellosis was 4.2% (95% CI: 1.5%−8.2%, 214/8,416, Table 3, Supplementary material S6, S7); after 2011, the prevalence of canine brucellosis was 3.1% (95% CI: 1.2%−6.0%, 281/11,472, Table 3, Supplementary material S6, S7), resulting in no significant differences between sampling years (*P* > 0.05).

### 3.4 Testing method risk factor assessment results

Canine brucellosis positivity was higher when tested using the Test strip method (14.1%; 95% CI: 10.8%−17.9%; 52/368, Table 3, Supplementary material S6, S7) than other testing methods, resulting in a significant difference between detection method (*P* < 0.05).

### 3.5 *Brucella* species types risk factor assessment results

On *Brucella* species type, *Brucella* roughened (R type, 3.1%; 95% CI: 1.4%−5.5%; 225/9,924, Table 3, Supplementary material S6, S7) is more positive than *Brucella* smooth (S type, 2.3%; 95% CI: 0.8%−4.5%;138/8,879, Table 3, Supplementary material S6, S7).

### 3.6 Gender risk factor assessment results

In terms of gender, males (2.2%; 95% CI: 1.6%−3.0%; 47/1,595, Table 3, Supplementary material S6, S7) were more likely to be positive for brucellosis than females (1.73% %; 95% CI: 1.1%−2.5%; 34/1,274, Table 3, Supplementary material S6, S7), but the difference was not significant (*P* > 0.05).

### 3.7 Age-related risk factor assessment results

In terms of age, dogs aged ≥3 years had the highest brucellosis positivity rate (2.2%; 95% CI: 0.1–3.9; 28/1,000, Table 3, Supplementary material S6, S7), with the prevalence of brucellosis increasing with age.

### 3.8 Husbandry method risk factor assessment results

Among different management practices, stray dogs exhibited the highest prevalence rate of 22.6% (95% CI: 18.3%−27.2%, Table 3, Supplementary material S6, S7), followed by domestic dog rate of 9.5% (95% CI: 5.4%−14.6%, Table 3, Supplementary material S6, S7). In contrast, breed dogs and outpatient dogs show lower prevalence rates of 5.1% (95% CI: 2.0%−9.4%, Table 3, Supplementary material S6, S7) and 5.2% (95% CI: 2.0%−10.0%, Table 3, Supplementary material S6, S7), respectively. Free-range dogs have the lowest prevalence of 0.8% (95% CI: 0.1%−2.0%, Table 3, Supplementary material S6, S7), indicating a significant variation in brucellosis prevalence across different dog management practices.

### 3.9 Geographical factors risk factor assessment results

We assessed the prevalence of brucellosis in dogs across various subgroups based on geographical and climatic factors, finding that the highest prevalence rates were associated with longitudes between 80°-100° (20.3%, 95% CI: 11.5%−30.8%, Table 5, Supplementary material S6, S7), latitudes between 40°-50° (20.3%, 95% CI: 11.0%−31.5%, Table 5, Supplementary material S6, S7), areas with 0–500 mm annual rainfall (17.4%, 95% CI: 6.6%−32.0%, Table 5, Supplementary material S6, S7), and low temperature regions, suggesting that these factors may contribute to the observed heterogeneity. Significant variation was observed with respect to latitude, longitude, rainfall, humidity, and temperature extremes. For instance, areas with moderate humidity (60%−70%) had a prevalence of 9.6% (95% CI: 0.3%−29.4%, Table 5, Supplementary material S6, S7), and regions experiencing temperatures of 5°C and below showed the highest brucellosis prevalence (31.4%, 95% CI: 21.3%−42.6%, Table 5, Supplementary material S6, S7). High heterogeneity was noted across all subgroups, with *I*^2^ values ranging from 77.0% to 97.1%, indicating substantial variability among studies.

**Table 5:** Geographical factors analysis of brucellosis in dog in China.

		**No. studies**	**No. tested**	**No. positive**	% (95%^*^CI)	**Heterogeneity**			**Univariate meta-regression**		**Correlation analysis**
						χ^2^	***P*** **-value**	*I*^2^ **(%)**	***P*** **-value**	**Coefficient (95% CI)**	*R* ^2^ **-region**
Latitude	20–30°	5	2,816	66	2.1% (0.7–4.1)	35.32	< 0.01	88.7%			61.52%
	30–40°	6	2,072	46	2.5% (0.6–5.6)	63.81	< 0.01	92.2%			
	40–50°	5	1,362	293	20.3% (11.0–31.5)	91.99	< 0.01	95.7%	< 0.0001	0.315 (0.212 to 0.418)	
Longitude	80–100°	3	965	204	20.3% (11.5–30.8)	28.45	< 0.01	93.0%	0.0014	0.266 (0.103 to 0.429)	12.21%
	100–110°	5	1,332	43	4.0% (0.5–10.8)	79.56	< 0.01	95.0%			
	110–125°	8	3,953	158	4.0% (11.5–30.8)	240.89	< 0.01	97.1%			
Rainfall	0–500 mm	5	1,060	247	17.4% (6.6–32.0)	122.76	< 0.01	96.7%	< 0.0001	0.313 (0.199 to 0.427)	54.34%
	500–1,000 mm	5	1,754	29	1.8% (0.3–4.6)	36.07	< 0.01	88.9%			
	1,000–3,000 mm	4	2,704	55	1.3% (0.4–2.8)	20.99	< 0.01	85.7%			
Humidity	35–60%	6	1,795	69	4.6% (0.8–11.3)	131.90	< 0.01	96.2%			37.70%
	60–70%	4	1,005	97	9.6% (0.3–29.4)	194.73	< 0.01	98.5%	0.351	0.101 (−0.111 to 0.312)	
	70–85%	5	2,718	165	4.3% (0.2–13.4)	278.85	< 0.01	98.6%			
Temperature	5–5°C	3	580	188	31.4% (21.3–42.6)	8.69	0.01	77.0%	< 0.0001	0.418 (0.269 to 0.567)	67.00%
	5–15°C	5	1,670	42	3.0% (0.4–7.8)	72.68	< 0.01	94.5%			
	15–25°C	6	3,268	101	2.8% (0.8–5.9)	94.59	< 0.01	94.7%			
Lowest temperature	−10 to 0°C	4	700	189	14.3% (1.3–37.8)	113.06	< 0.01	97.3%	0.0022	0.251 (0.090 to 0.411)	23.78%
	0–10°C	4	1,550	41	3.7% (0.4–10.1)	72.18	< 0.01	95.8%			
	10–20°C	6	3,268	101	2.8% (0.8–5.9)	94.59	< 0.01	94.7%			
Highest temperature	0–10°C	2	577	188	33.6% (25.5–42.2)	4.29	0.04	76.7%	< 0.0001	0.451 (0.300 to 0.602)	68.57%
	10–20°C	5	1,673	42	3.0% (0.4–7.8)	72.27	< 0.01	94.5%			
	20–30°C	6	3,268	101	2.8% (0.8–5.9)	94.59	< 0.01	94.7%			

^*^CI, confidence interval; R^2^, proportion of between-study variance explained by joint test with provinces as a covariate.

### 3.10 Publication bias and sensitivity analysis results

**Figure 4:**
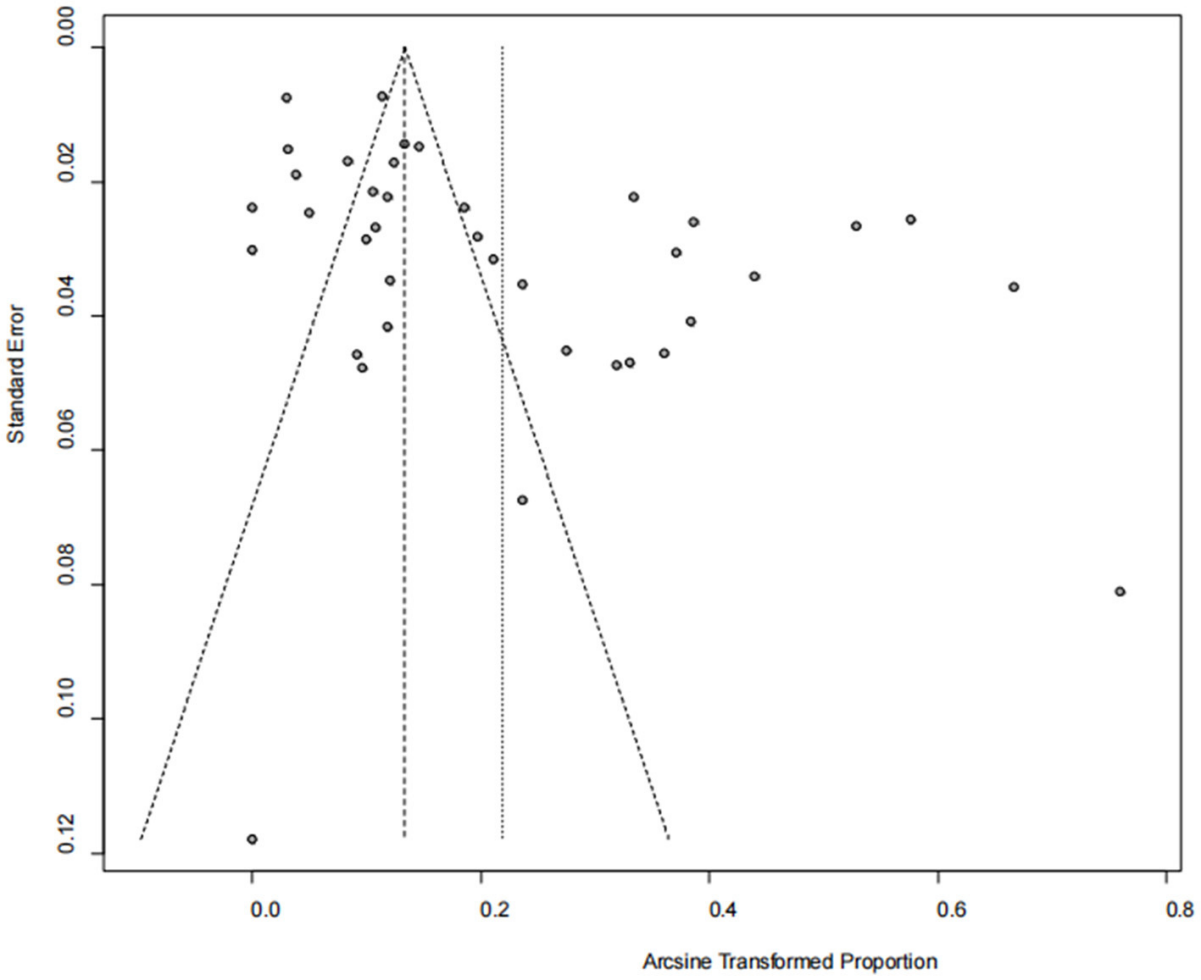
Funnel plot with pseudo 95% confidence limits interval for the examination of publication bias.

**Figure 5:**
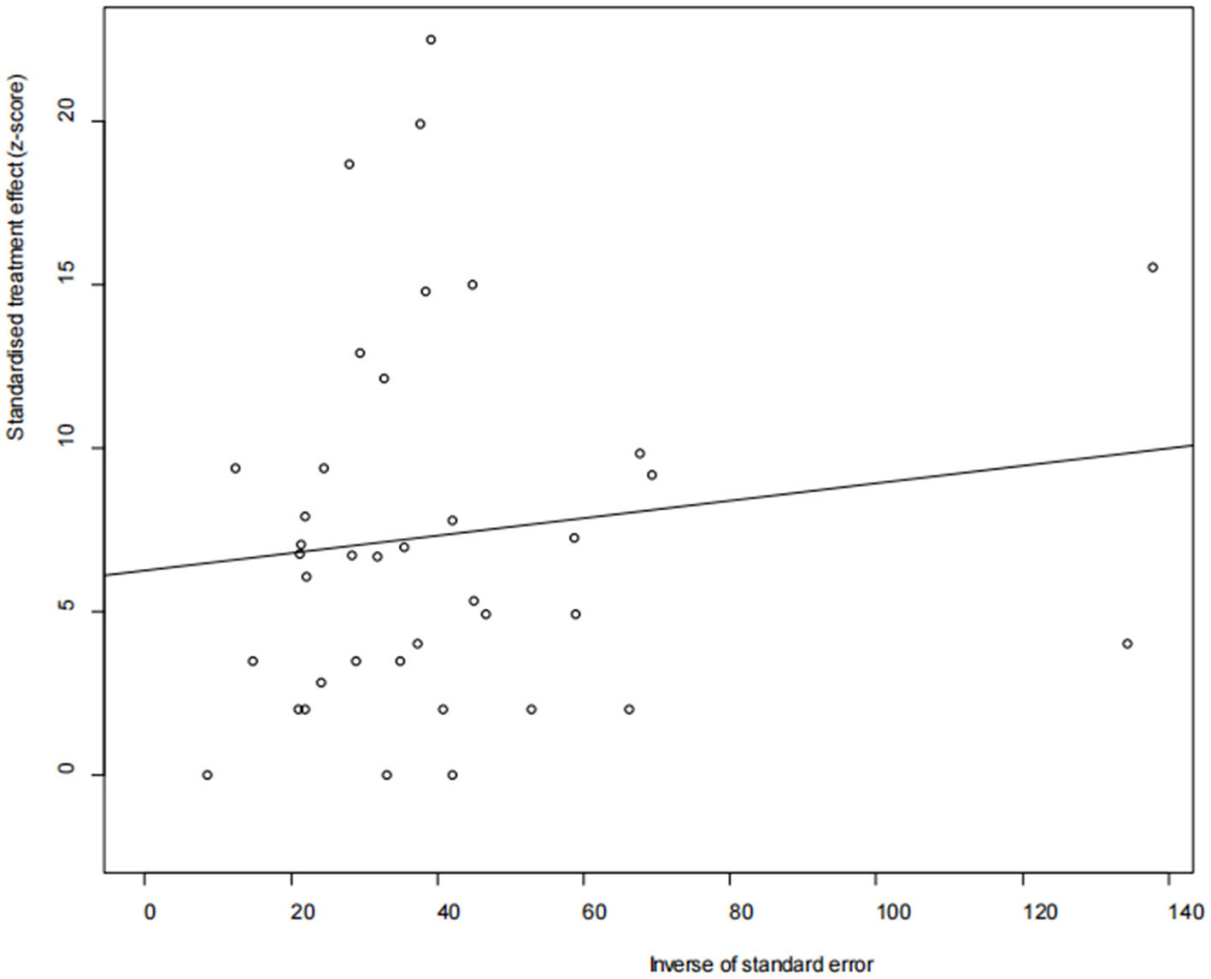
Egger's test for publication bias.

**Figure 6:**
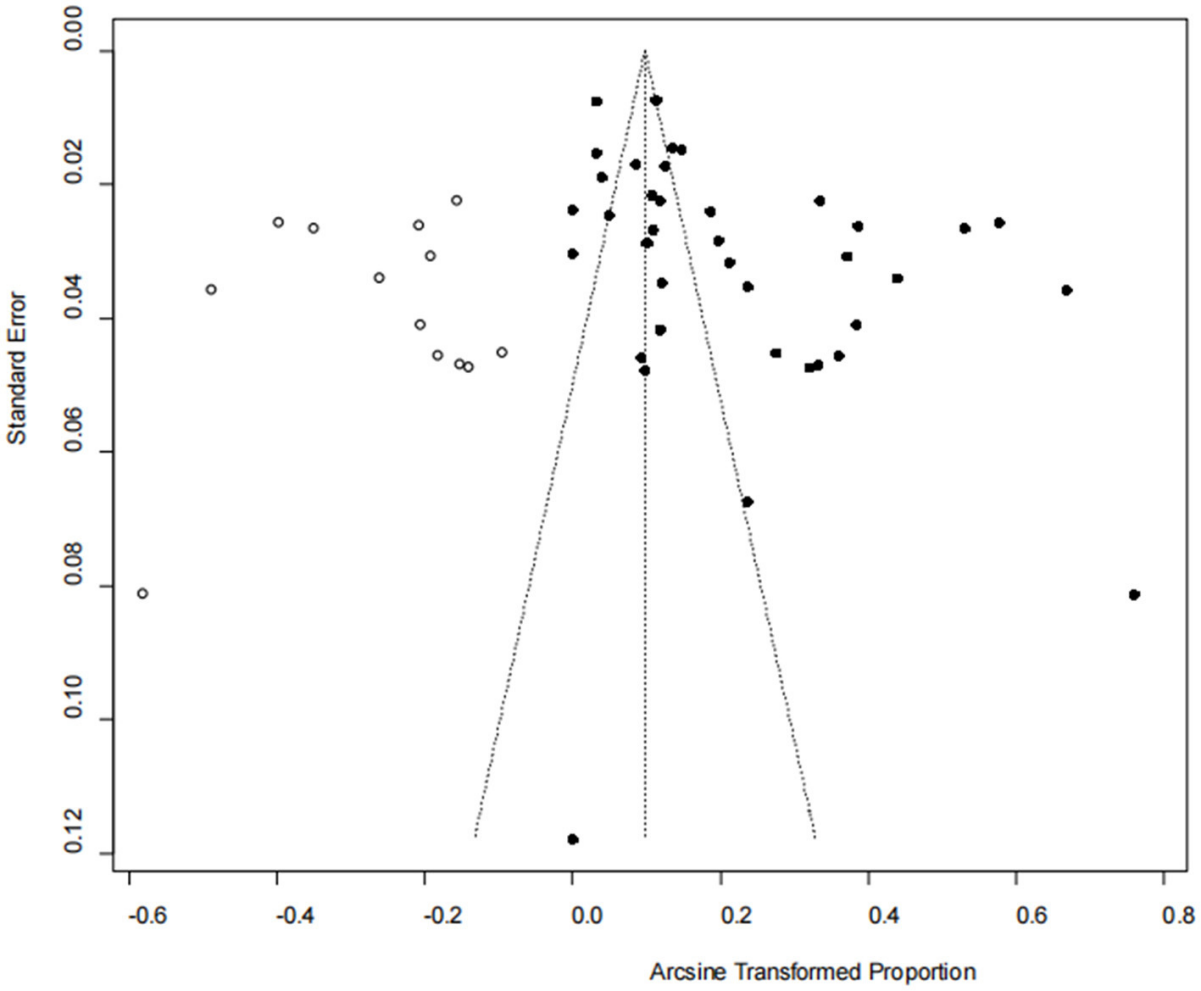
Trim-and-fill chart to detect research bias.

**Figure 7:**
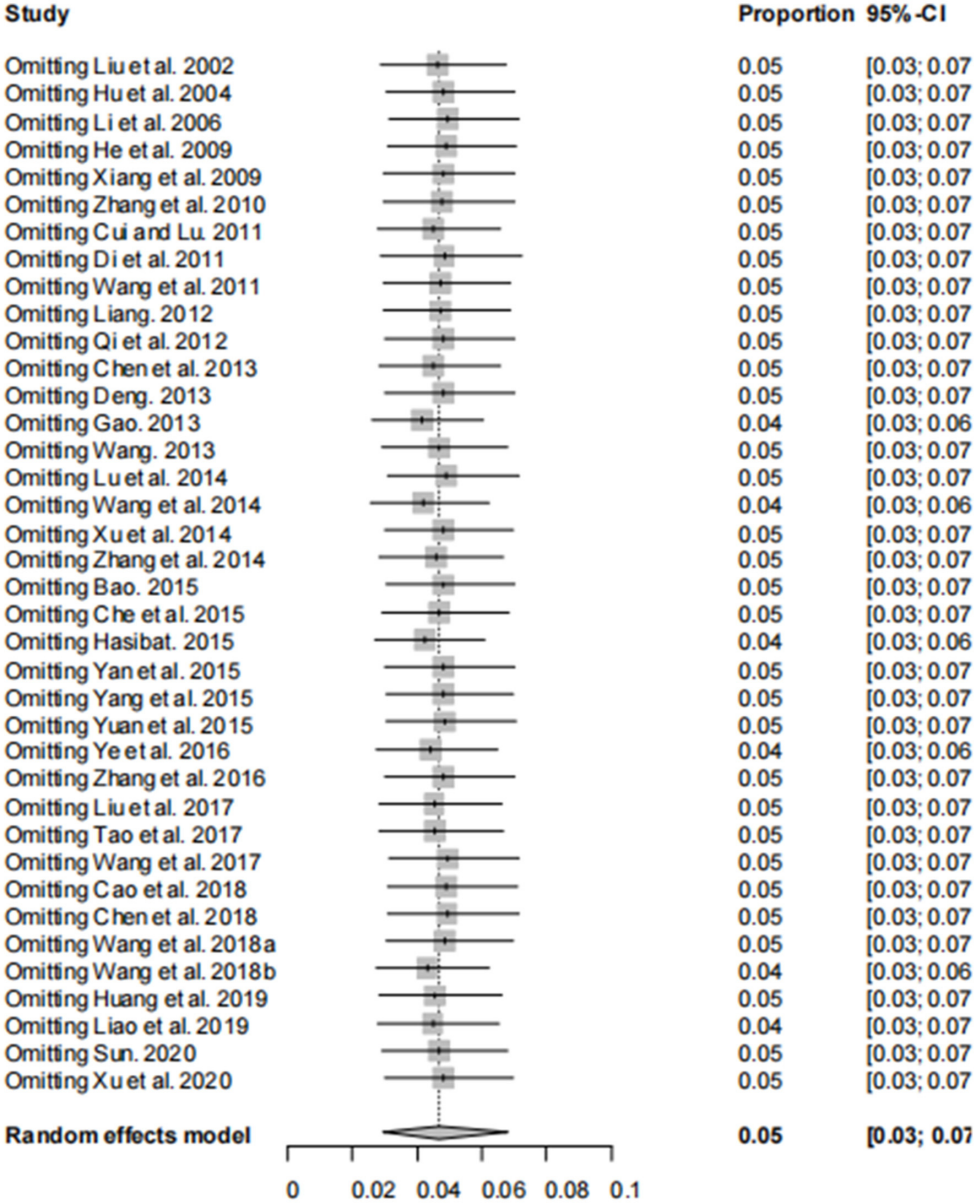
Sensitivity analysis, after excluding one study, the results of other studies were not significantly reversed.

The asymmetry observed in the funnel plot suggests the possibility of publication bias or small study effects (Figure 4). However, Egger's linear regression test (*P* = 0.001) did not indicate any significant publication bias (Figure 5). This finding was further supported by the trim-and-fill analysis. This analysis demonstrated that the impact of publication bias was minimal, confirming the robustness of the study results (Figure 6). Additionally, the forest plot and the *I*^2^ statistic indicated a high degree of heterogeneity among the studies (*I*^2^ = 97.7%, *P* **<** 0.01; Figure 2). Sensitivity analysis, performed by excluding individual studies and reanalyzing the remaining data. This revealed no substantial changes in the overall results, indicating that the findings are robust and credible (Figure 7).

## 4 Discussion

Canines are susceptible to infection with *Brucella*, a bacterium that can cause disease in dogs, pigs, cattle, and sheep (50). In canines, the disease can manifest as abortions in females, reddened and swollen penises in males, enlarged testicles, epididymitis, and reduced fertility (3). It has been demonstrated that *Brucella* can persist for an extended period in some dogs that have recovered from the disease. These animals can excrete, defecate and secrete large numbers of pathogenic bacteria. It is therefore evident that sick animals and long-term carriers represent the main source of infection (27). The advancement of productivity and socio-economic development in contemporary society has resulted in an improvement in living standards, accompanied by an intensification of the workload for younger individuals and an increase in the number of empty nesters, which has led to a rise in pet ownership. As significant companion animals, pet dogs are in close contact with humans, resulting in the frequent occurrence of canine-induced brucellosis in humans (4, 10). It is therefore of great importance to analyze the incidence and risk factors associated with canine brucellosis, with a view to safeguarding human and public health.

The first isolation of *Brucella canis* occurred in China in 1984. By 1989, the presence of *Brucella canis* infections in dogs, humans and other animals had been reported, with varying degrees of severity (6). In China, the prevalence of canine brucellosis infection was more significant prior to the 1990s (6), with further cases reported since 2000 (44). The 2007–2009 canine brucellosis survey demonstrated a notable decline in the infection rate compared to the 1980s. However, the positive rate of canine brucellosis exhibited an upward trend following 2000, coinciding with an increase in inter-animal brucellosis outbreaks (45). In recent years, an increasing number of cases of canine brucellosis infection have been reported, indicating the urgent need for the prevention and control of canine brucellosis (28, 50). The effect of sampling year on canine brucellosis was analyzed. It was found that the incidence of canine brucellosis decreased slightly after 2010 in comparison to before 2010. This is not consistent with the above findings. This discrepancy can be attributed to the fact that during the period spanning 2012 to 2016, a total of 4,508 canine serum samples were tested in the Animal Brucellosis Seropositivity Survey Report of Ivana City. However, the number of positive serum samples among them was only 4, representing a positive rate of 0.09% (44). The sample size of dogs after 2010 in this survey was total 11,472, so the above test report greatly reduces the positive rate of canine brucellosis after 2010.

In recent years, there has been a notable increase in the number of cases of canine brucellosis infection reported in various provinces and cities. A prevalence analysis of canine brucellosis in different provinces and cities revealed a significantly higher prevalence in Inner Mongolia and the Xinjiang Autonomous Region compared to other provinces and cities. This finding is consistent with the observed trend of inter-animal brucellosis outbreaks. Furthermore, research has shown that canine brucellosis has been reported in 23 provinces (municipalities) since 1989, with a prevalence rate reaching up to 38.87% in Hulun Buir City, Inner Mongolia (6, 26). Notably, China's four major pastoral regions include Inner Mongolia, Xinjiang, Tibet, and Qinghai. Of these, Inner Mongolia and Xinjiang are the largest pastoral areas in China. They have cattle and sheep herds that are considerably larger than those of other provinces and cities. As a consequence of the expansion of pasture areas, the density of cattle, sheep and other economic livestock rearing has increased. This has also led to an increase in the rearing of sheepdogs. The close contact between sheepdogs, cattle and sheep and other livestock in these environments has resulted in a higher rate of infection with brucellosis in canines than in agricultural and urban areas (26). There is also a potential public health risk due to the complexity of *Brucella* species infecting dogs in pastoral areas and the presence of *Brucella melitensis*, a smooth type that is highly pathogenic to humans (6). We propose to increase publicity and education to improve the understanding of brucellosis among shepherds; to reasonably regulate the stocking density of cattle, sheep and other domestic animals to avoid excessive stocking density leading to the spread of brucellosis in the population; to keep shepherd dogs and cattle, sheep and other domestic animals separate and to prohibit the feeding of abortions and deliveries of cattle and sheep to avoid cross-infection; paying attention to the environmental hygiene of the feeding area and disinfecting it regularly; improving the nutritional value of the feeds and the appropriate combination of the dogs' diets to enhance immunity; regular vaccination of livestock, including cattle and sheep, and serological testing of cattle, sheep, and shepherd dogs are essential measures. Furthermore, enhanced rodent control efforts are necessary to minimize the risk of *Brucella* transmission through rodents.

When we analyzed for different regions, we found that there was no significant difference in the positive rate of canine brucellosis. It's indicating that the occurrence of canine brucellosis is a local phenomenon. Therefore, in China, canine brucellosis is predominantly sporadically disseminated. Genetic diversity analyses of 63 strains of *Brucella canis* isolated in China between 1983 and 2011 also confirmed that there were no significant molecular epidemiological associations with cases from other regions. Furthermore, the results demonstrated that *Brucella canis* did not cause nationwide epidemics or outbreaks of brucellosis. Instead, outbreaks occurred only in more concentrated dog breeding bases (51, 52). In particular, three small-scale outbreaks of canine brucellosis occurred in an experimental dog breeding base in 2011, which were found to be caused by the introduction of infected dogs in conjunction with on-site epidemiological investigations, suggesting that the introduction of infected dogs may be an important cause of canine brucellosis outbreaks (29). Therefore, *Brucella* in our dog breeds is characterized by a geographical origin in which imported and Chinese-specific lineages co-exist. It is recommended that increased vigilance be exercised when introducing new breeds and that quarantine testing be intensified. It is also recommended that sick and infected dogs be promptly removed from the population. Following the introduction of new breeds, it is essential to implement comprehensive isolation measures, closed management, meticulous disinfection procedures, and strict avoidance of contact with domestic animals. Additionally, the introduction of dogs into the breeding population should only occur after a thorough observation period, during which time the breeding management should be strengthened.

We further analyzed the classification of *Brucella* and found no significant difference in infection rates between the R-type and S-type strains, indicating that canine brucellosis is not only caused by *Brucella canis*, but that infections with other species of *Brucella* are also present. The presence of *Brucella* R and S was observed in experimental dogs in Guangdong (41), and the same *Brucella* species were identified in pet hospitals and kennels in Chengdu (49). Additionally, serological testing of stray dogs revealed the co-infection of *Brucella* Rough and *Brucella* Smooth, indicating the potential for interspecies transmission of *Brucella*. Further investigation is required to elucidate the precise mechanism of infection. In one study, *Brucella* R was isolated from dogs in the Xinjiang region. Anti-*Brucella* antibodies were detected in sera from dairy cows, production ewes and breeding rams, and a strain of *Brucella canis* was isolated from cattle, suggesting possible host transfer for canine brucellosis in China (36). A comparable canine infection with smooth brucellosis is observed in numerous instances within China, for example: From 2010 to 2018, nine cities in China have reported a succession of cases of disseminated canine brucellosis (5). These include 13 cases in Dalian (53), 8 cases in Xinxiang (54), 3 cases in Loudi (55), and 11 cases in Kaifeng (56). A single case was identified in Dongguan (57) and Huai'an (58), with several cases related to the feeding of sheep scraps. Additionally, cases were reported in Dandong (59), Fushun (60), and Urumqi (61). The aforementioned studies have demonstrated that canine brucellosis presents with a mixed infection of type R and type S, and that interspecies and host transfer of *Brucella* can occur, which is consistent with the findings of this study.

The analysis of subgroups of assays revealed significant differences between the methods employed. The test strip tests exhibited the highest positivity rate, followed by the ELISA and RBPT methods. In contrast, the SAT and PCR methods demonstrated the lowest rates of detection. *Brucella canis* is a rough type of the bacteria, yet has the same cell membranes as *Brucella abortus, Brucella melitensis* and *Brucella suis* (39). As a result, the diagnosis of canine brucellosis is a relatively complex process. Firstly, it should be noted that serological tests are highly sensitive when performed on the surface antigens of these bacteria, however, they are not particularly specific (23), resulting in a high false-positive rate for the serological detection of brucellosis in dogs. Furthermore, chronic cases of brucellosis in dogs may yield negative results, necessitating the complementation of serological tests with pathogenic bacteriological studies. Despite some limitations in the use of serological methods for the diagnosis of canine brucellosis, these techniques can be valuable in the screening process. Additionally, two serological tests have been employed: the indirect fluorescent antibody test (IFA) and the enzyme-linked immunosorbent assay (ELISA). However, the sensitivity of IFA is unreliable, and some infected dogs cannot be diagnosed using this method. Furthermore, ELISA demonstrated greater specificity than IFA, with the ability to detect positive samples as early as 30 days post-infection in dogs (62). Secondly, the process of bacterial culture is inherently time-consuming and necessitates the expertise of trained professionals. In the case of blood cultures, the process of obtaining bacterial cultures from animals with relatively large sampling requirements who have been treated with antibiotics can be more challenging. Furthermore, samples such as aborted fetuses, semen, vaginal secretions, lymph nodes, bone marrow, and urine are frequently employed for the purpose of bacterial culture. Given the slow growth rate of this bacterium and the distinctive characteristics of the bacteria in question, a negative culture result may not be sufficient to definitively exclude the possibility of infection. In the event that the sample in question lacks an adequate quantity of live bacteria, the likelihood of a negative bacterial culture result is high. However, the possibility of the presence of the bacteria in the sick dog remains. The positive results of the Tiger Red plate agglutination test were significantly higher than those of the tube agglutination test in the present study, indicating that the Tiger Red plate agglutination test exhibited a high level of false positives. This may be attributed to cross-reactivity of the serological tests, given that the affected dogs were infected with other bacteria. It can be observed that the tiger red plate agglutination test is only applicable as a screening test, necessitating the utilization of a more specific diagnostic tool to confirm the diagnosis. Polymerase chain reaction (PCR) is a molecular biology method for the detection of *Brucella canis*, including both live and dead organisms. The DNA of *Brucella canis* can be detected using PCR (47). Semen, vaginal secretions, uterine excretions and urine are all suitable for PCR analysis. Whole blood may also be employed as a test sample; however, PCR is not advised in the early stages due to the requisite duration of illness for the establishment of bacteremia. Therefore, we suggest that serological tests combined with molecular biology tests or pathogen diagnostic methods can be used as the best method for the combined diagnosis of canine brucellosis, which may improve accuracy.

*Brucella canis* is an intracellular pathogenic bacterium that primarily affects steroid-producing tissues, including the testes, epididymis, prostate, and uterus of dogs (39). A number of routes facilitate the excretion/secretion of pathogenic bacteria, including semen, urine (males are particularly susceptible), female dog births, abortions, fetuses, placentas, malodor, vaginal secretions, urine, and milk (47). A sex subgroup analysis revealed that the prevalence of male dogs was slightly higher than that of female dogs, indicating that *Brucella canis* can primarily transmitted through mating, genital contact, aborted fetuses, placenta, and vaginal secretions. It is also noteworthy that cages, equipment and individuals who have been in contact with infected canines may represent additional sources of infection. It is therefore recommended that all dogs should undergo serological testing prior to mating, at least once a year, and that new animals entering kennels should be placed under quarantine and tested to ensure their health status. It is recommended that sick and pregnant dogs be kept separate from healthy dogs, that they be quarantined regularly, and that kennels and utensils be sterilized on a regular basis.

The analysis of age subgroups revealed that canines over 3 years of age exhibited a higher prevalence of *Brucella* than those aged 1–3 years and those under 1 year. Although this difference did not reach statistical significance, the findings indicate that there are age-related differences in susceptibility to *Brucella* in dogs. Puppies have an immature immune system and enter estrus at ~1 year of age, a period that increases the risk of *Brucella* infection during breeding. Conversely, at 3 years of age, dogs enter their reproductive prime, exhibiting alterations in physiology and immune status, which coincide with a peak in susceptibility to *Brucella* (19, 22). It is therefore recommended that farms adopt a strategy of self-breeding in order to avoid cross-infection among different groups of dogs. This approach can effectively reduce the risk of *Brucella* infections during sexual maturity and the peak breeding season. This approach will contribute to the safeguarding of the overall health and breeding quality of dogs. Furthermore, farms should implement differentiated preventive measures for dogs of different ages, and reinforce daily management and regular health monitoring to ensure that the health status of the entire dog population at all ages is effectively maintained, thus providing a safer and healthier breeding environment for the dogs.

Following an analysis of the categorization of the dogs, significant differences in the incidence of brucellosis were identified between the different categories of dogs. The prevalence of brucellosis in stray dogs is significantly higher than that of other types of dogs. This may be attributed to the complexity of their living environment, which includes uncertain food sources, frequent outdoor activities and a lack of health management. These factors may thus increase the probability of exposure to and infection with *Brucella*. The prevalence of disease in domesticated dogs is higher than in breeding dogs, primarily due to their exposure to outdoor environments, increased likelihood of contact with infectious agents, and reduced concern for disease prevention compared to specialized breeders. Conversely, breeder populations on farms exhibit a lower prevalence rate, which can be attributed to the fact that they are typically self-bred, thereby reducing the invasion of external pathogens. Additionally, they are often reared in a more stable living environment and subject to strict health management. These measures collectively serve to reduce the incidence of brucellosis. The disparity in positivity rates can be attributed to several factors. Primarily, the manner in which the canines are maintained plays a pivotal role. Canines sourced from settings other than commercial dog farms are typically kept in free-range conditions, with intricate living arrangements and regular interaction with other domesticated animals, including cows, sheep, and pigs. Secondly, the diet of some of the dogs was also complex, with the practice of feeding them raw mutton, beef, pork, cattle and sheep runoff and placenta. Furthermore, dog owners demonstrate a lack of awareness regarding canine brucellosis, as well as a deficiency in knowledge pertaining to its prevention and control. The aforementioned circumstances introduce an element of uncertainty with regard to the prevention and control of canine brucellosis. In light of the aforementioned data, it is this author's recommendation that distinct health management strategies be developed in accordance with the characteristics of the various breeds and sources of dogs. Based on these data, we propose to formulate targeted health management strategies according to the characteristics of different dog breeds and sources. Strengthening the regulation of stray dogs. It is recommended that breeding dogs on farms should continue to be kept in an optimal breeding environment and maintained to ensure overall herd health and population stability. It is recommended that domesticated dogs undergo regular medical check-ups, avoid consuming raw meat and coming into contact with brucellosis-infected dogs, breed safely and seek timely medical treatment to ensure the health and safety of domesticated dogs.

The incidence of canine brucellosis demonstrates a notable increase during the spring and summer months, coinciding with the rise in temperature. During this period, dogs are more active and have more contact with other animals, thus increasing the risk of infection with the *Brucella* bacteria. Furthermore, the elevated humidity levels characteristic of the spring and summer months create more conducive conditions for the survival and reproduction of *Brucella*. Air with higher humidity maintains the viability of the bacteria, enabling them to persist longer in the environment and thus increasing the probability of infection in dogs. During such seasons, dog owners are advised to exercise particular care with regard to the hygiene management of their dogs and to avoid contact with animals that may be carriers of *Brucella*, including sheep, cows and pigs. Concurrently, kennels must be meticulously cleaned and disinfected on a routine basis, utilizing efficacious disinfectants such as 1% sodium hypochlorite and 70% ethanol to curtail the survival and dissemination of the pathogen. Furthermore, dog owners are advised to enhance their observation of their dogs. In the event of the emergence of any unusual symptoms, such as fever, lethargy, joint discomfort, and so forth, it is imperative that the affected animal be promptly taken to a veterinary facility for medical evaluation and brucellosis testing. In addition to seasonal attention, long-term preventive measures should be established for the prevention and control of canine brucellosis. This encompasses the implementation of routine health assessments on canines, particularly serological testing, and the continuous observation and maintenance of the kennel environment. The implementation of these comprehensive preventive and control measures will effectively reduce the incidence of canine brucellosis, thereby safeguarding the health and safety of both dogs and humans.

This meta-analysis offers the benefits of a long timeframe, extensive scope, and robust analytical techniques, but it is not without limitations. First, the language of the included studies was restricted to English or Chinese, meaning studies in other languages might have been missed. Second, the articles were selected from only six databases, which could have excluded relevant studies from other sources. Third, the amount of information available from the included studies was sometimes insufficient, potentially introducing publication bias or other forms of bias within the analyzed subgroups. Fourth, some risk factors were assessed in a limited number of studies and samples, which may have led to a small study effect and produced small stable results. Additionally, the study included only a partial representation of provinces in the Central China region, with some provinces lacking relevant reports altogether. The absence of articles from these provinces may lead to inaccurate estimations of the prevalence of canine brucellosis in the region.

In summary, canine brucellosis is widely distributed across China, with particularly high prevalence rates in pastoral areas. The high prevalence of canine brucellosis not only results in significant economic losses for herders but also increases the risk of human brucellosis infection. Therefore, it is recommended to implement long-term surveillance in pastoral regions, control breeding density, and establish appropriate isolation measures between dogs and livestock to reduce the transmission rate. Considering that human contact with dogs can increase the risk of brucellosis infection, we suggest regular vaccination, avoiding close contact with dogs, and refraining from consuming raw meat to mitigate the infection risk. Additionally, in regions where canine brucellosis is not given adequate attention, it is essential to enhance public awareness and education, and conduct epidemiological investigations as soon as possible to provide a theoretical basis for brucellosis control.

## Data Availability

The original contributions presented in the study are included in the article/Supplementary material, further inquiries can be directed to the corresponding authors.
