# Supplementary material for: Prevalence of Brucella in dogs in China: a systematic review and meta-analysis—Epidemiological analysis of canine brucellosis
Source: Front Vet Sci. 2025 Feb 13;11:1515405. doi: 10.3389/fvets.2024.1515405 (PMC11866426; doi:10.3389/fvets.2024.1515405)
Supplement: Supplementary file 1 [file Data_Sheet_1.zip › Supporting information/S5. Strategy for searching.docx]

S1 Appendix.

Strategy for searching PubMed

((((((((((((((((((“Brucellosis”[Mesh]) OR (Brucellosis)) OR (Malta Fever)) OR (Fever, Malta)) OR (Gibraltar Fever)) OR (Fever, Gibraltar)) OR (Rock Fever)) OR (Fever, Rock)) OR (Cyprus Fever)) OR (Fever, Cyprus)) OR (*Brucella* Infection)) OR (*Brucella* Infections)) OR (Infection, *Brucella*)) OR (Undulant Fever)) OR (Fever, Undulant)) OR (Brucellosis, Pulmonary)) OR (Brucellosis, Pulmonary)) OR (Pulmonary Brucellosis)) OR (Pulmonary Brucellosis)) AND (((“Dogs”[Mesh]) OR (Dog)) OR (Canis familiaris)) AND ((((((“China” [Mesh]) OR (People's Republic of China)) OR (Mainland China)) OR (Manchuria)) OR (Sinkiang)) OR (Inner Mongolia)).

Strategy for searching Spring link and Science Direct

Search for (Dogs and Brucellosis and Prevalence)

Strategy for searching Wan fang, VIP, and CNKI

Search for (Dog (in Chinese) and Brucellosis (in Chinese))
