# Supplementary material for: Prevalence of Brucella in dogs in China: a systematic review and meta-analysis—Epidemiological analysis of canine brucellosis
Source: Front Vet Sci. 2025 Feb 13;11:1515405. doi: 10.3389/fvets.2024.1515405 (PMC11866426; doi:10.3389/fvets.2024.1515405)
Supplement: Supplementary file 1 [file Data_Sheet_1.zip › Supporting information/S6. Forest map.pdf]

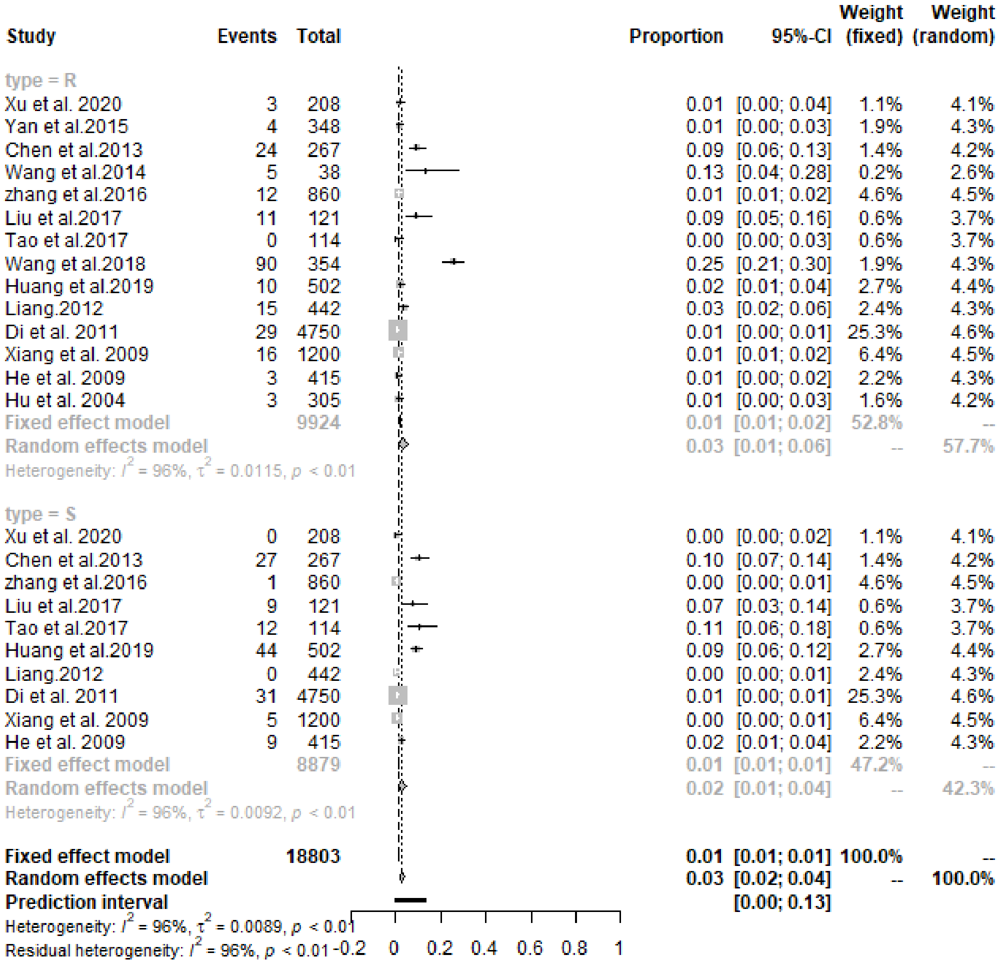

Figure S1. Forest plot of prevalence of the *Brucella* in dogs for *Brucella* type

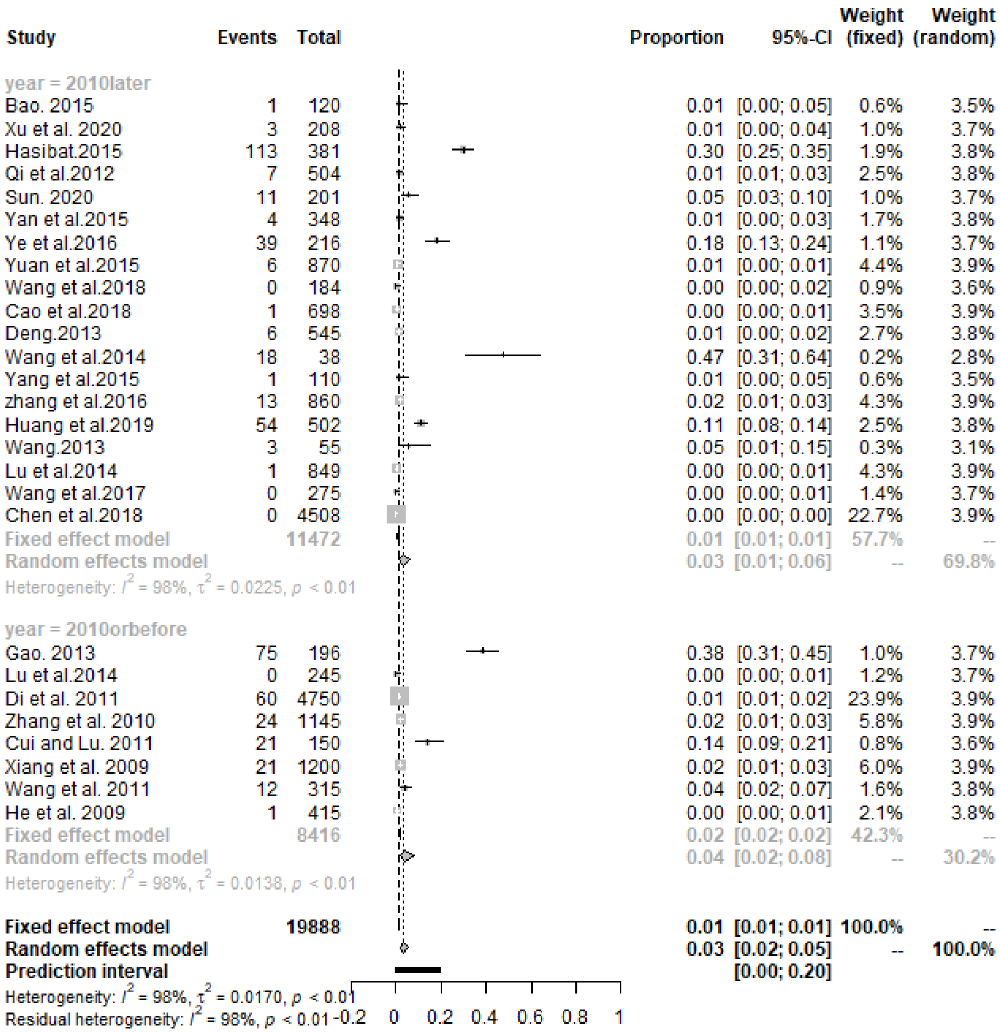

Figure S2. Forest plot of prevalence of the *Brucella* in dogs for sampling year

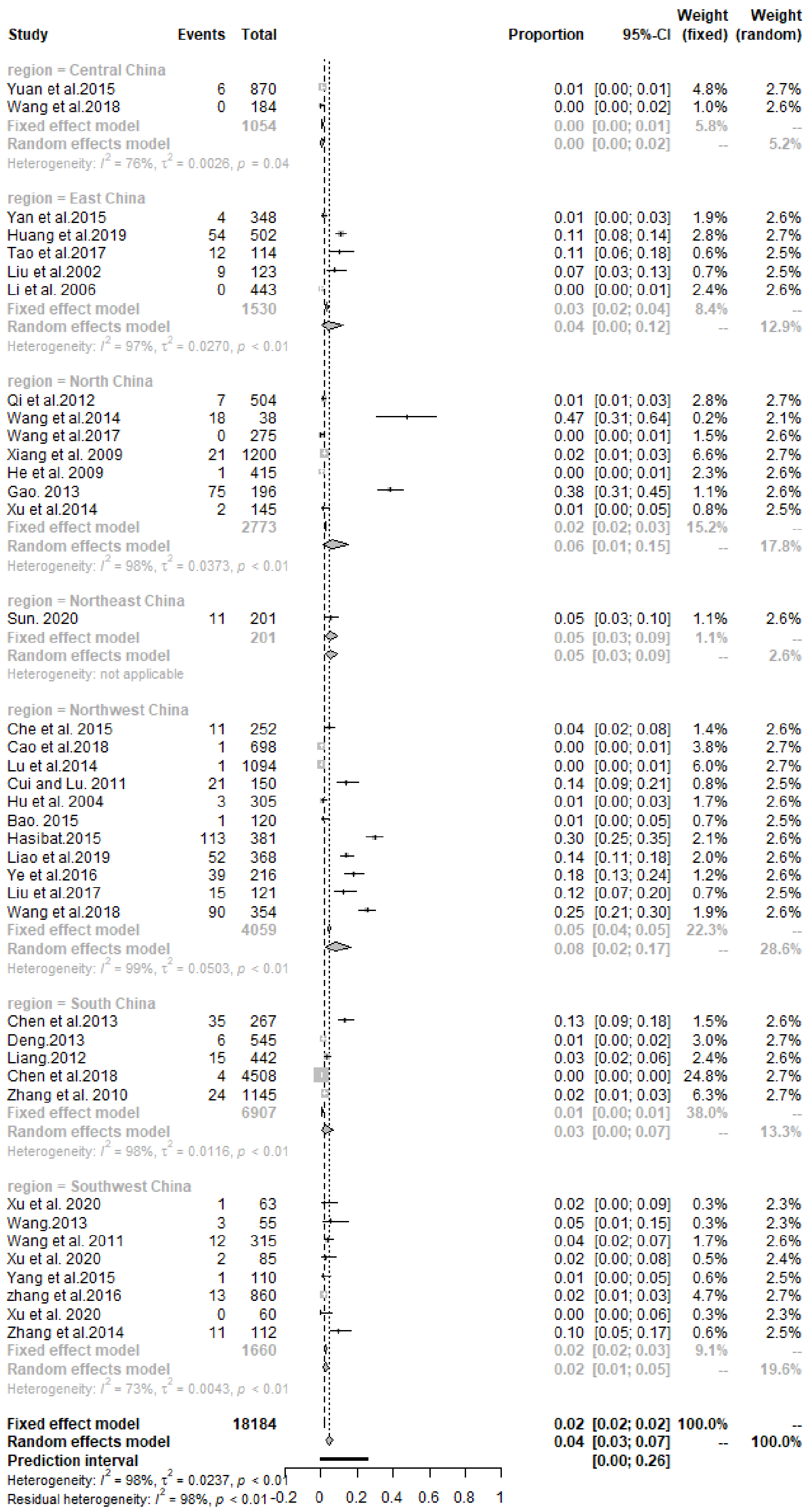

Figure S3. Forest plot of prevalence of the *Brucella* in dogs for region

Study	Events	Total	Proportion	95%-CI	Weight (fixed)	Weight (random)
method = ELISA						
Ye et al.2016	39	216	0.18	[0.13; 0.24]	0.9%	2.7%
Yuan et al.2015	6	870	0.01	[0.00; 0.01]	3.8%	2.8%
Liu et al.2017	15	121	0.12	[0.07; 0.20]	0.5%	2.6%
Wang et al.2018	90	354	0.25	[0.21; 0.30]	1.6%	2.8%
Huang et al.2019	54	502	0.11	[0.08; 0.14]	2.2%	2.8%
Fixed effect model		2063	0.07	[0.06; 0.08]	9.1%	--
Random effects model			0.12	[0.03; 0.26]	--	13.7%
Heterogeneity: $I^2 = 98\%$, $\tau^2 = 0.0419$, $p < 0.01$						
method = PCR						
Sun. 2020	11	201	0.05	[0.03; 0.10]	0.9%	2.7%
Zhang et al.2014	11	112	0.10	[0.05; 0.17]	0.5%	2.6%
Wang et al.2017	0	275	0.00	[0.00; 0.01]	1.2%	2.7%
Fixed effect model		588	0.02	[0.01; 0.03]	2.6%	--
Random effects model			0.03	[0.00; 0.14]	--	8.0%
Heterogeneity: $I^2 = 95\%$, $\tau^2 = 0.0280$, $p < 0.01$						
method = RBPT						
Bao. 2015	1	120	0.01	[0.00; 0.05]	0.5%	2.6%
Hasibat.2015	113	381	0.30	[0.25; 0.35]	1.7%	2.8%
Fixed effect model		501	0.20	[0.16; 0.23]	2.2%	--
Random effects model			0.11	[0.00; 0.52]	--	5.3%
Heterogeneity: $I^2 = 99\%$, $\tau^2 = 0.1160$, $p < 0.01$						
method = SAT						
Che et al. 2015	11	252	0.04	[0.02; 0.08]	1.1%	2.7%
Xu et al. 2020	3	208	0.01	[0.00; 0.04]	0.9%	2.7%
Gao. 2013	75	196	0.38	[0.31; 0.45]	0.9%	2.7%
Qi et al.2012	7	504	0.01	[0.01; 0.03]	2.2%	2.8%
Yan et al.2015	4	348	0.01	[0.00; 0.03]	1.5%	2.8%
Cao et al.2018	1	698	0.00	[0.00; 0.01]	3.1%	2.8%
Chen et al.2013	35	267	0.13	[0.09; 0.18]	1.2%	2.7%
Deng.2013	6	545	0.01	[0.00; 0.02]	2.4%	2.8%
Wang et al.2014	18	38	0.47	[0.31; 0.64]	0.2%	2.1%
Xu et al.2014	2	145	0.01	[0.00; 0.05]	0.6%	2.6%
Yang et al.2015	1	110	0.01	[0.00; 0.05]	0.5%	2.6%
zhang et al.2016	13	860	0.02	[0.01; 0.03]	3.8%	2.8%
Tao et al.2017	12	114	0.11	[0.06; 0.18]	0.5%	2.6%
Wang.2013	3	55	0.05	[0.01; 0.15]	0.2%	2.3%
Lu et al.2014	1	1094	0.00	[0.00; 0.01]	4.8%	2.8%
Liang.2012	15	442	0.03	[0.02; 0.06]	1.9%	2.8%
Chen et al.2018	4	4508	0.00	[0.00; 0.00]	19.8%	2.9%
Di et al. 2011	60	4750	0.01	[0.01; 0.02]	20.9%	2.9%
Zhang et al. 2010	24	1145	0.02	[0.01; 0.03]	5.0%	2.8%
Cui and Lu. 2011	21	150	0.14	[0.09; 0.21]	0.7%	2.6%
Xiang et al. 2009	21	1200	0.02	[0.01; 0.03]	5.3%	2.8%
Wang et al. 2011	12	315	0.04	[0.02; 0.07]	1.4%	2.7%
He et al. 2009	1	415	0.00	[0.00; 0.01]	1.8%	2.8%
Liu et al.2002	9	123	0.07	[0.03; 0.13]	0.5%	2.6%
Li et al. 2006	0	443	0.00	[0.00; 0.01]	1.9%	2.8%
Hu et al. 2004	3	305	0.01	[0.00; 0.03]	1.3%	2.7%
Fixed effect model		19230	0.01	[0.01; 0.01]	84.5%	--
Random effects model			0.03	[0.02; 0.05]	--	70.2%
Heterogeneity: $I^2 = 96\%$, $\tau^2 = 0.0099$, $p < 0.01$						
method = Test strip						
Liao et al.2019	52	368	0.14	[0.11; 0.18]	1.6%	2.8%
Fixed effect model		368	0.14	[0.11; 0.18]	1.6%	--
Random effects model			0.14	[0.11; 0.18]	--	2.8%
Heterogeneity: not applicable						
**Fixed effect model**		**22750**	**0.02**	**[0.02; 0.02]**	**100.0%**	--
**Random effects model**			**0.05**	**[0.03; 0.07]**	--	**100.0%**
**Prediction interval**				**[0.00; 0.24]**		
Heterogeneity: $I^2 = 98\%$, $\tau^2 = 0.0191$, $p < 0.01$						
Residual heterogeneity: $I^2 = 97\%$, $p < 0.01$						

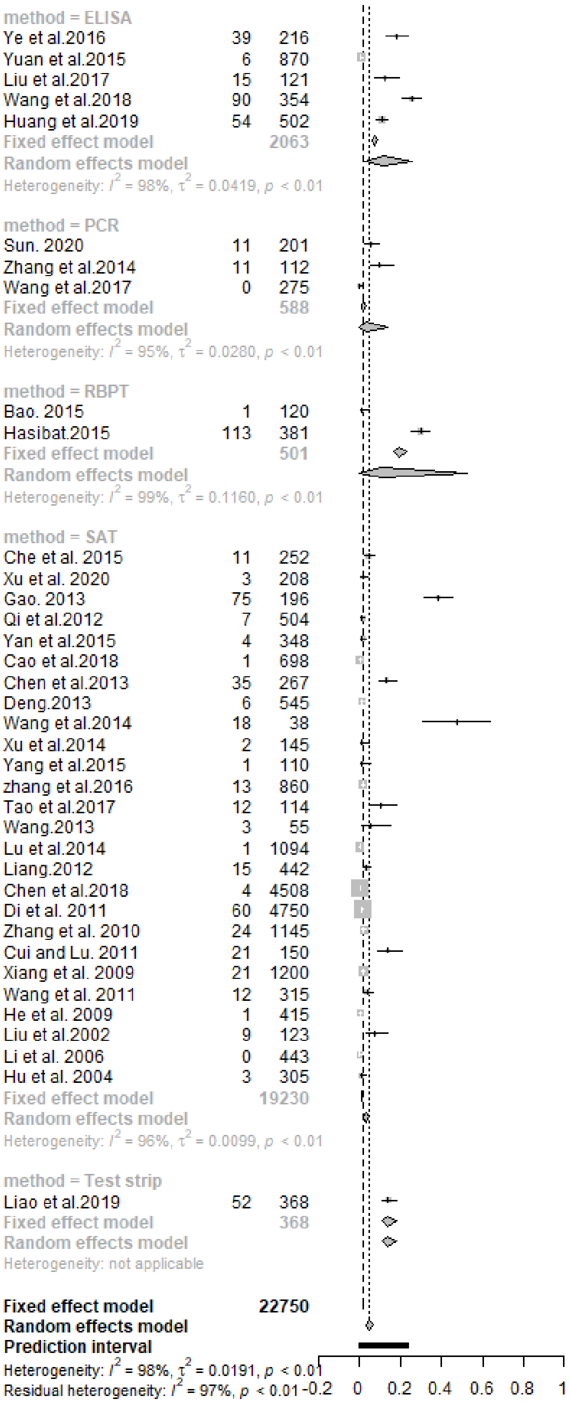

Figure S4. Forest plot of prevalence of the *Brucella* in dogs for detection method

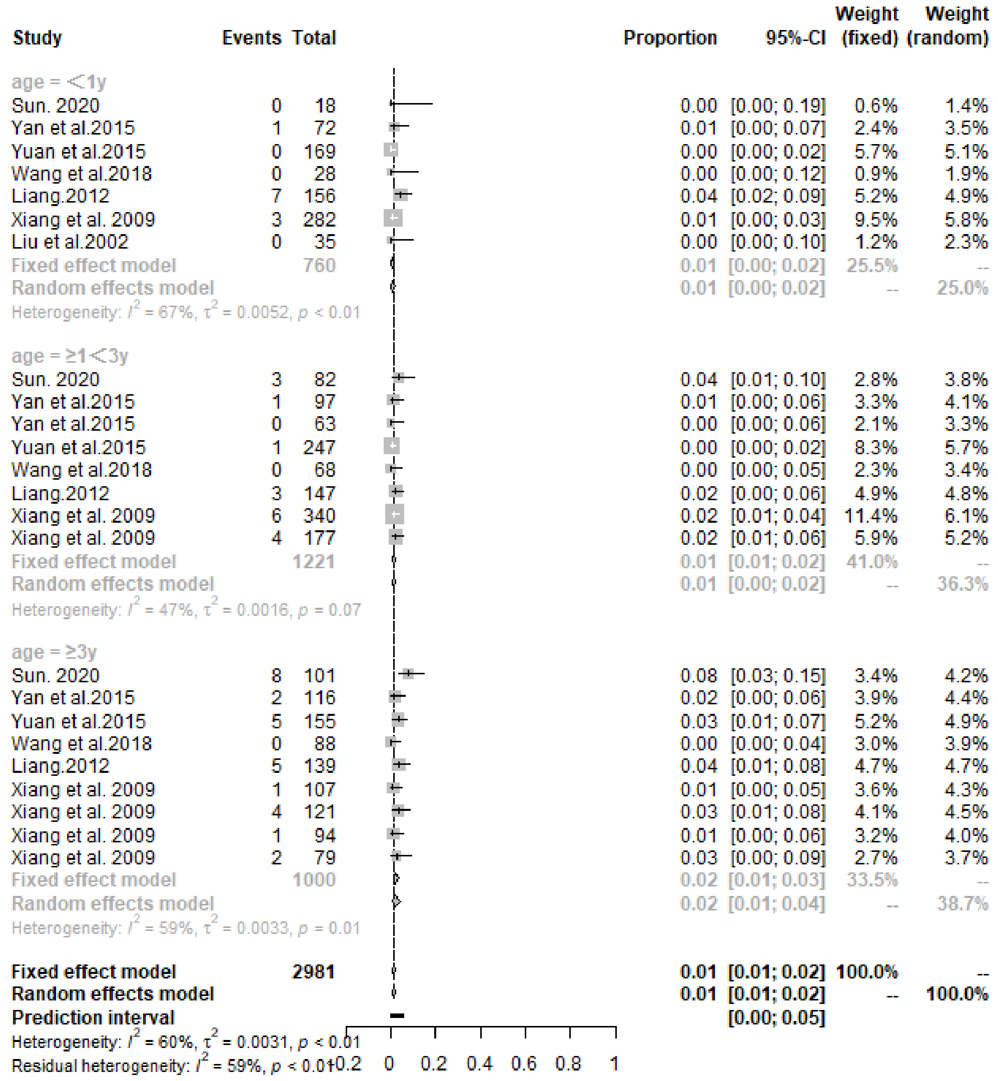

Figure S5. Forest plot of prevalence of the *Brucella* in dogs for age

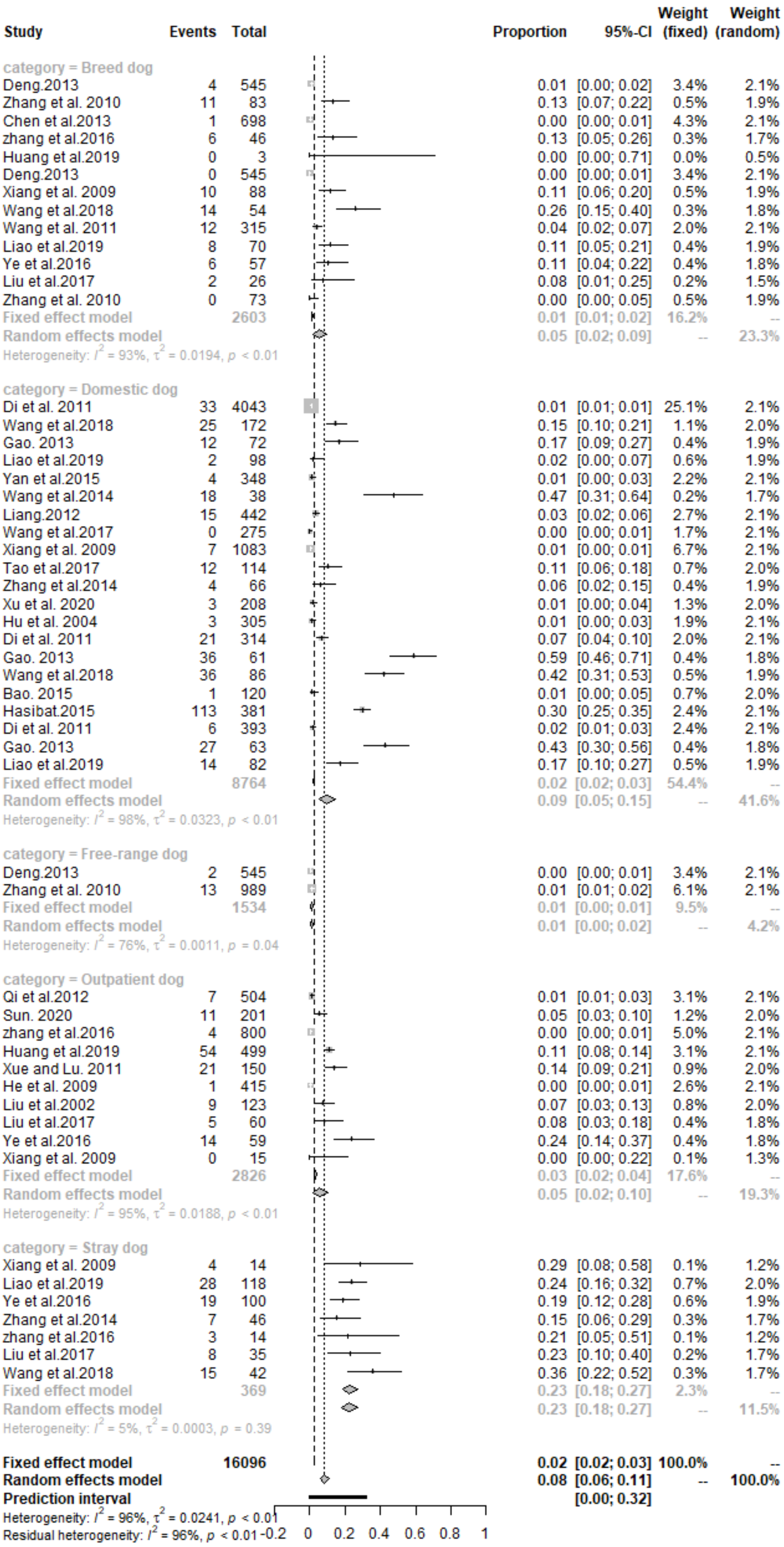

Figure S6. Forest plot of prevalence of the *Brucella* in dogs for farming mode

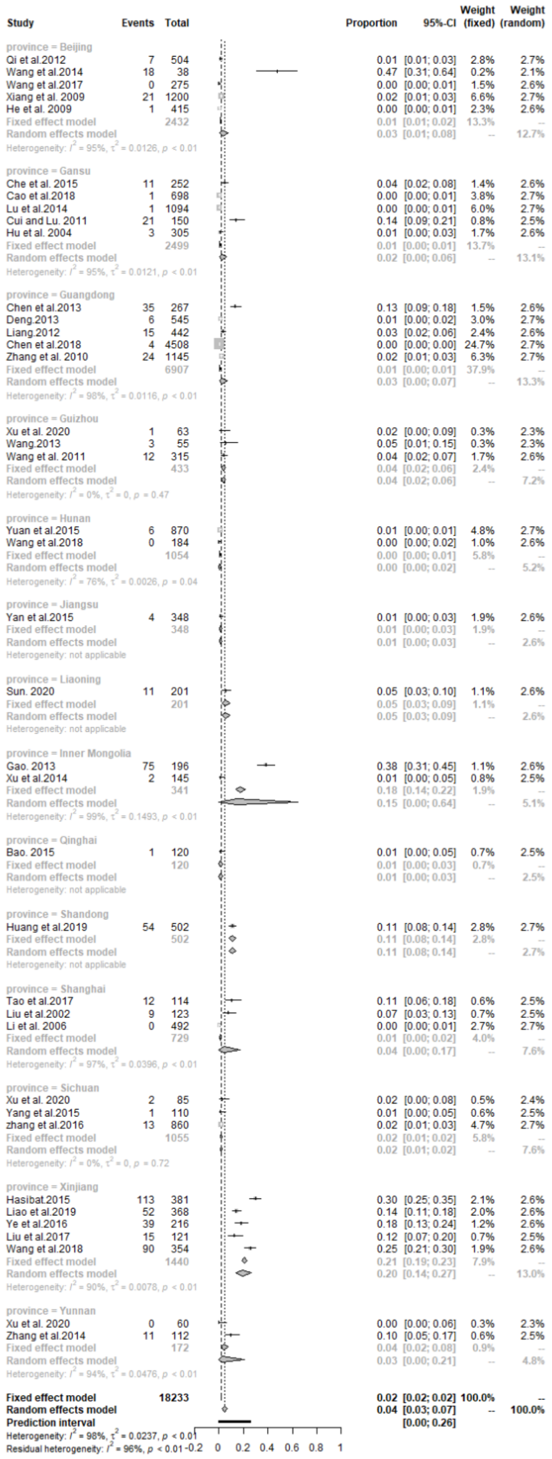

Figure S7. Forest plot of prevalence of the *Brucella* in dogs for province

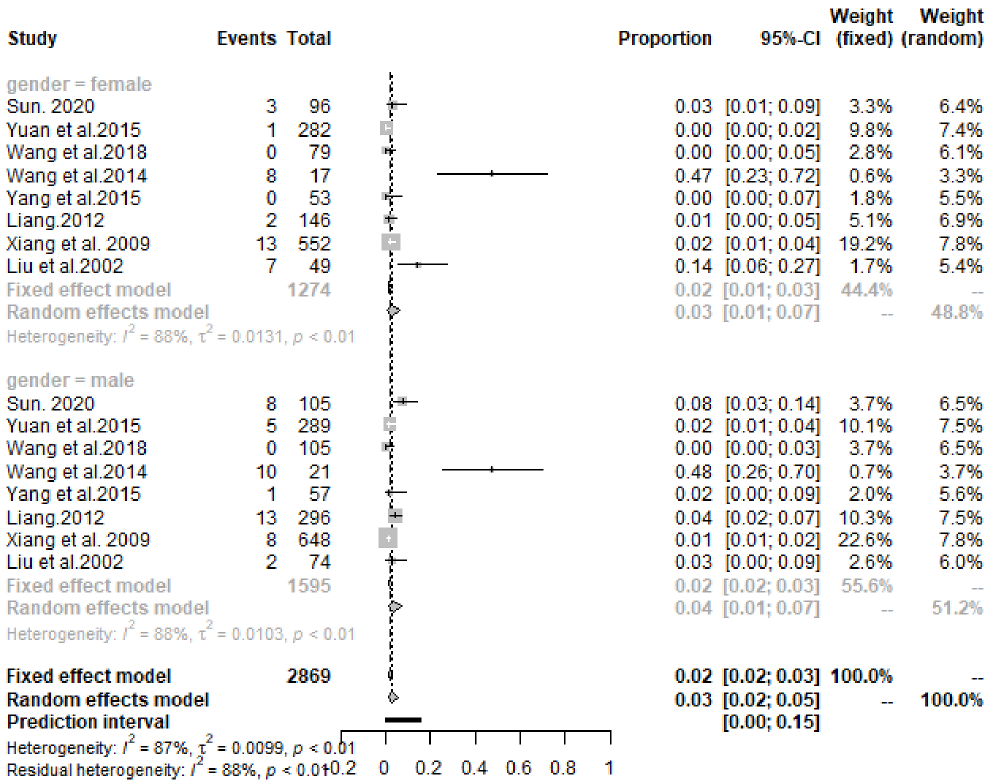

Figure S8. Forest plot of prevalence of the *Brucella* in dogs for gender

Study	Events	Total	Proportion	95%-CI	Weight (fixed)	Weight (random)
score = 0-1						
Li et al. 2006	0	443	0.00	[0.00; 0.01]	1.9%	2.7%
Fixed effect model		443	0.00	[0.00; 0.00]	1.9%	--
Random effects model			0.00	[0.00; 0.00]	--	2.7%
Heterogeneity: not applicable						
score = 2-3						
Zhang et al.2014	11	112	0.10	[0.05; 0.17]	0.5%	2.5%
Wang et al.2018	0	18	0.00	[0.00; 0.19]	0.1%	1.6%
Chen et al.2013	35	267	0.13	[0.09; 0.18]	1.2%	2.7%
Huang et al.2019	54	502	0.11	[0.08; 0.14]	2.2%	2.7%
Liang.2012	15	442	0.03	[0.02; 0.06]	1.9%	2.7%
Wang et al. 2011	12	315	0.04	[0.02; 0.07]	1.4%	2.7%
Hu et al. 2004	3	305	0.01	[0.00; 0.03]	1.3%	2.7%
Che et al. 2015	11	252	0.04	[0.02; 0.08]	1.1%	2.7%
Gao. 2013	75	196	0.38	[0.31; 0.45]	0.9%	2.6%
Liao et al.2019	52	368	0.14	[0.11; 0.18]	1.6%	2.7%
Yan et al.2015	4	348	0.01	[0.00; 0.03]	1.5%	2.7%
Ye et al.2016	39	216	0.18	[0.13; 0.24]	0.9%	2.7%
Cao et al.2018	1	698	0.00	[0.00; 0.01]	3.1%	2.8%
Deng.2013	6	545	0.01	[0.00; 0.02]	2.4%	2.8%
Wang et al.2014	18	38	0.47	[0.31; 0.64]	0.2%	2.1%
Xu et al.2014	2	145	0.01	[0.00; 0.05]	0.6%	2.6%
Yang et al.2015	1	110	0.01	[0.00; 0.05]	0.5%	2.5%
zhang et al.2016	13	860	0.02	[0.01; 0.03]	3.8%	2.8%
Liu et al.2017	15	121	0.12	[0.07; 0.20]	0.5%	2.5%
Tao et al.2017	12	114	0.11	[0.06; 0.18]	0.5%	2.5%
Wang et al.2018	90	354	0.25	[0.21; 0.30]	1.6%	2.7%
Wang.2013	3	55	0.05	[0.01; 0.15]	0.2%	2.3%
Lu et al.2014	1	1094	0.00	[0.00; 0.01]	4.8%	2.8%
Chen et al.2018	4	4508	0.00	[0.00; 0.00]	19.8%	2.8%
Di et al. 2011	60	4750	0.01	[0.01; 0.02]	20.9%	2.8%
Zhang et al. 2010	24	1145	0.02	[0.01; 0.03]	5.0%	2.8%
Xiang et al. 2009	21	1200	0.02	[0.01; 0.03]	5.3%	2.8%
He et al. 2009	1	415	0.00	[0.00; 0.01]	1.8%	2.7%
Liu et al.2002	9	123	0.07	[0.03; 0.13]	0.5%	2.5%
Fixed effect model		19616	0.02	[0.01; 0.02]	86.2%	--
Random effects model			0.05	[0.03; 0.08]	--	75.9%
Heterogeneity: I^2 = 98%, τ^2 = 0.0171, p < 0.01						
score = 4-5						
Bao. 2015	1	120	0.01	[0.00; 0.05]	0.5%	2.5%
Hasibat.2015	113	381	0.30	[0.25; 0.35]	1.7%	2.7%
Qi et al.2012	7	504	0.01	[0.01; 0.03]	2.2%	2.7%
Sun. 2020	11	201	0.05	[0.03; 0.10]	0.9%	2.6%
Yuan et al.2015	6	870	0.01	[0.00; 0.01]	3.8%	2.8%
Wang et al.2017	0	275	0.00	[0.00; 0.01]	1.2%	2.7%
Cui and Lu. 2011	21	150	0.14	[0.09; 0.21]	0.7%	2.6%
Xu et al. 2020	3	208	0.01	[0.00; 0.04]	0.9%	2.6%
Fixed effect model		2709	0.03	[0.03; 0.04]	11.9%	--
Random effects model			0.04	[0.00; 0.11]	--	21.4%
Heterogeneity: I^2 = 98%, τ^2 = 0.0387, p < 0.01						
**Fixed effect model**		**22768**	**0.02**	**[0.02; 0.02]**	**100.0%**	**--**
**Random effects model**			**0.05**	**[0.03; 0.07]**	**--**	**100.0%**
**Prediction interval**				**[0.00; 0.23]**		
Heterogeneity: I^2 = 98%, τ^2 = 0.0191, p < 0.01						
Residual heterogeneity: I^2 = 98%, p < 0.01						

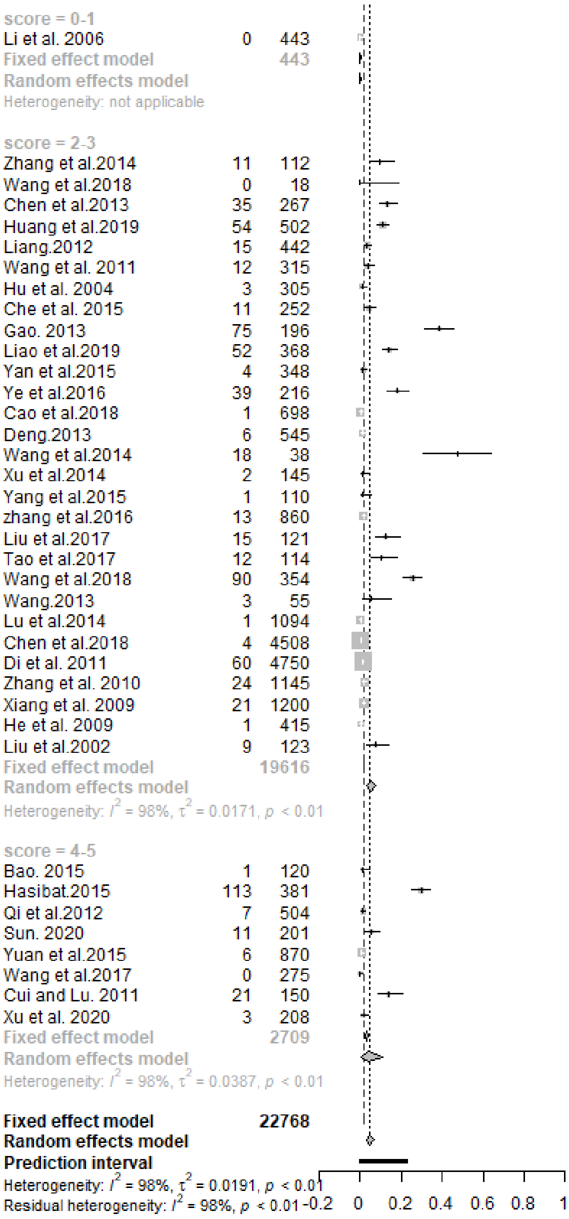

Figure S9. Forest plot of prevalence of the *Brucella* in dogs for quality

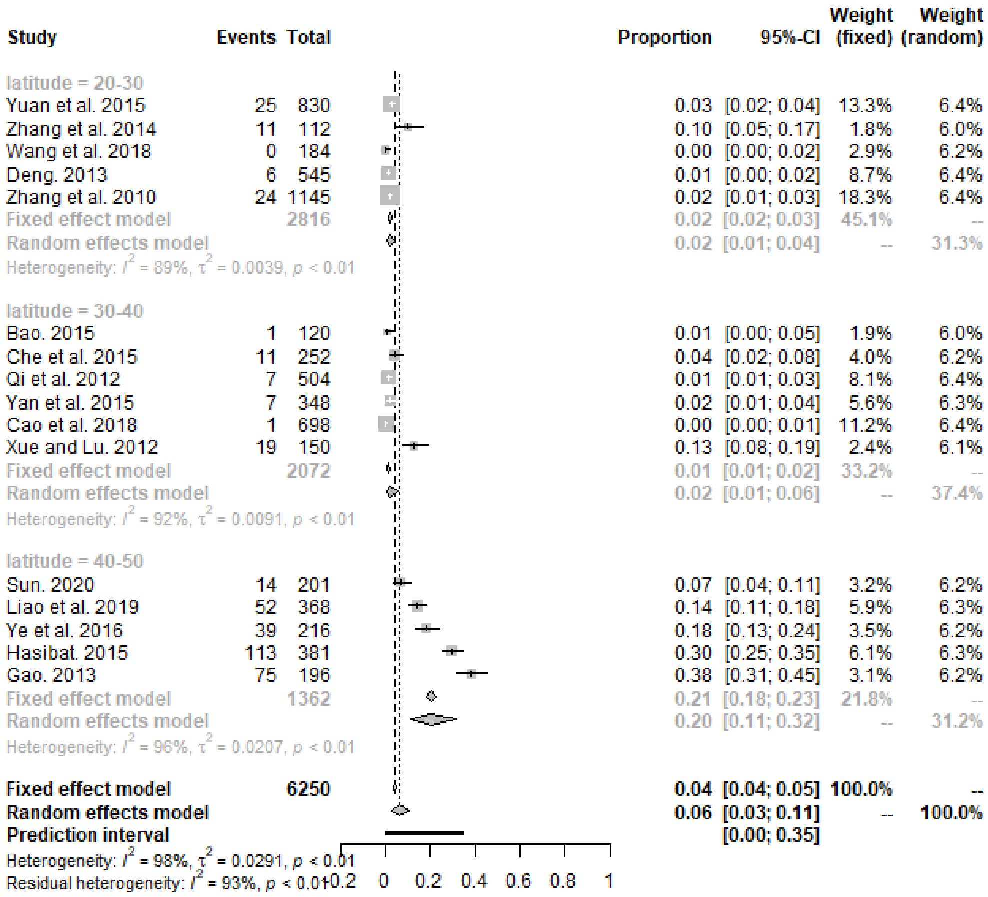

Figure S10. Forest plot of prevalence of the *Brucella* in dogs for Northern Latitude

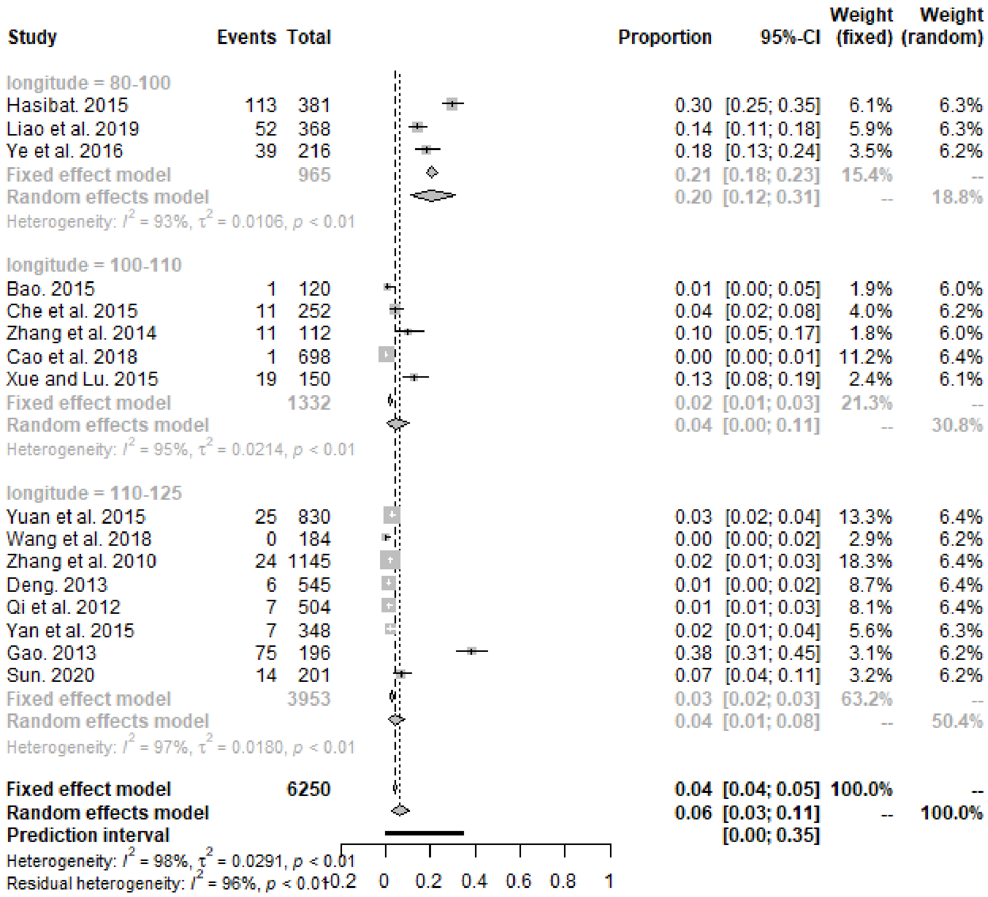

Figure S11. Forest plot of prevalence of the *Brucella* in dogs for Eastern Longitude

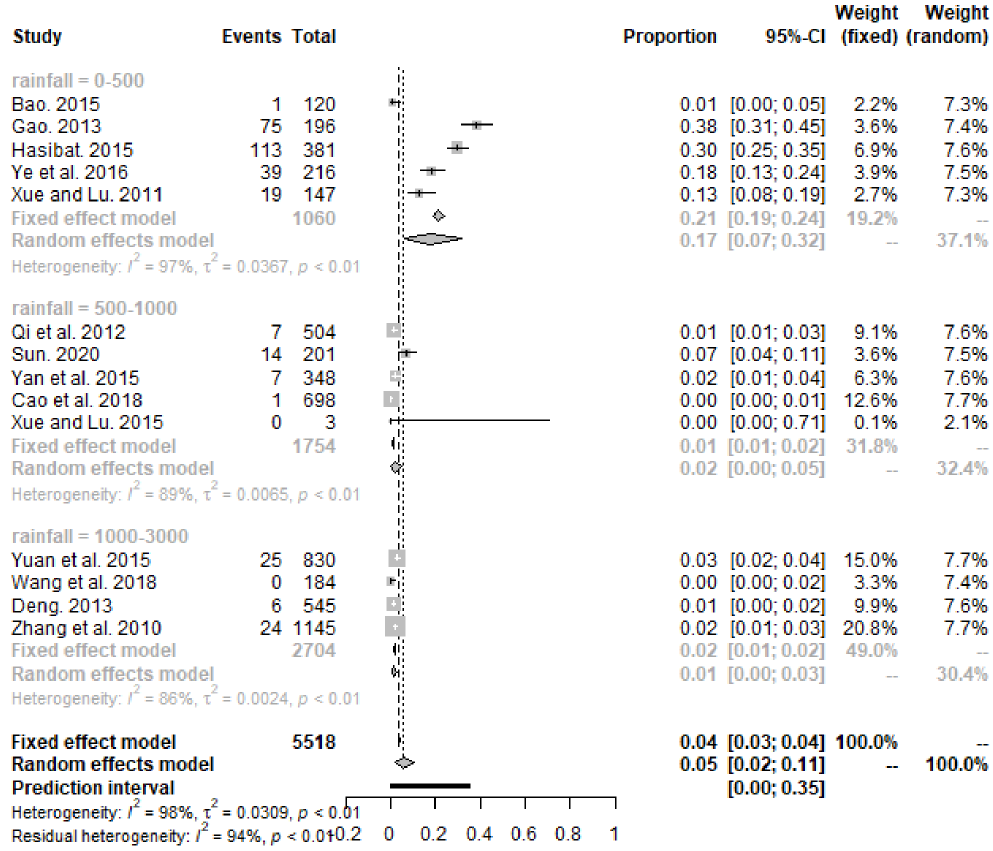

Figure S12. Forest plot of prevalence of the *Brucella* in dogs for Rainfall

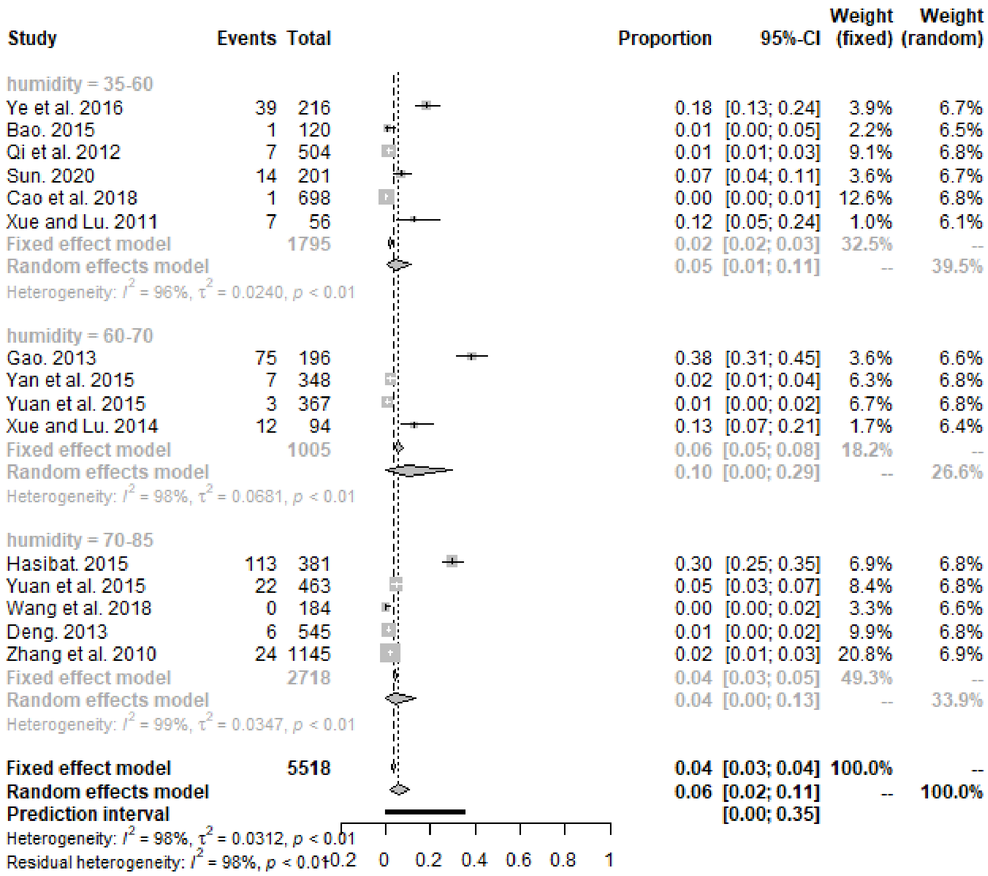

Figure S13. Forest plot of prevalence of the *Brucella* in dogs for humidity

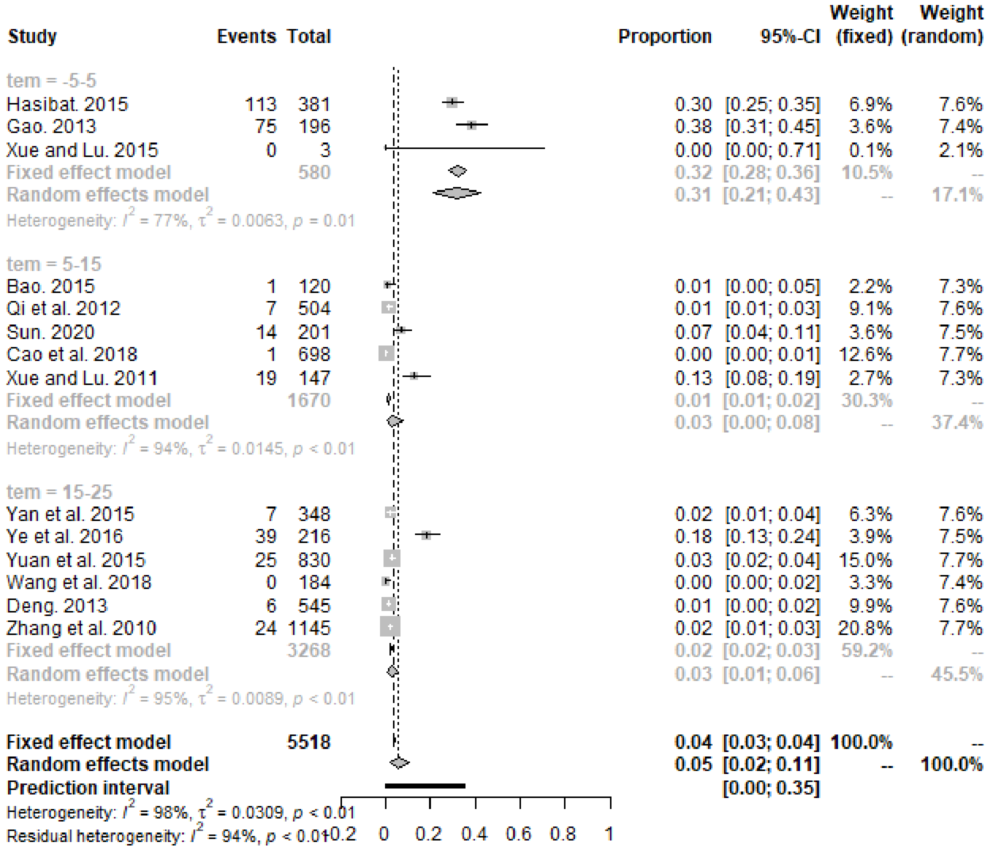

Figure S14. Forest plot of prevalence of the *Brucella* in dogs for temperature

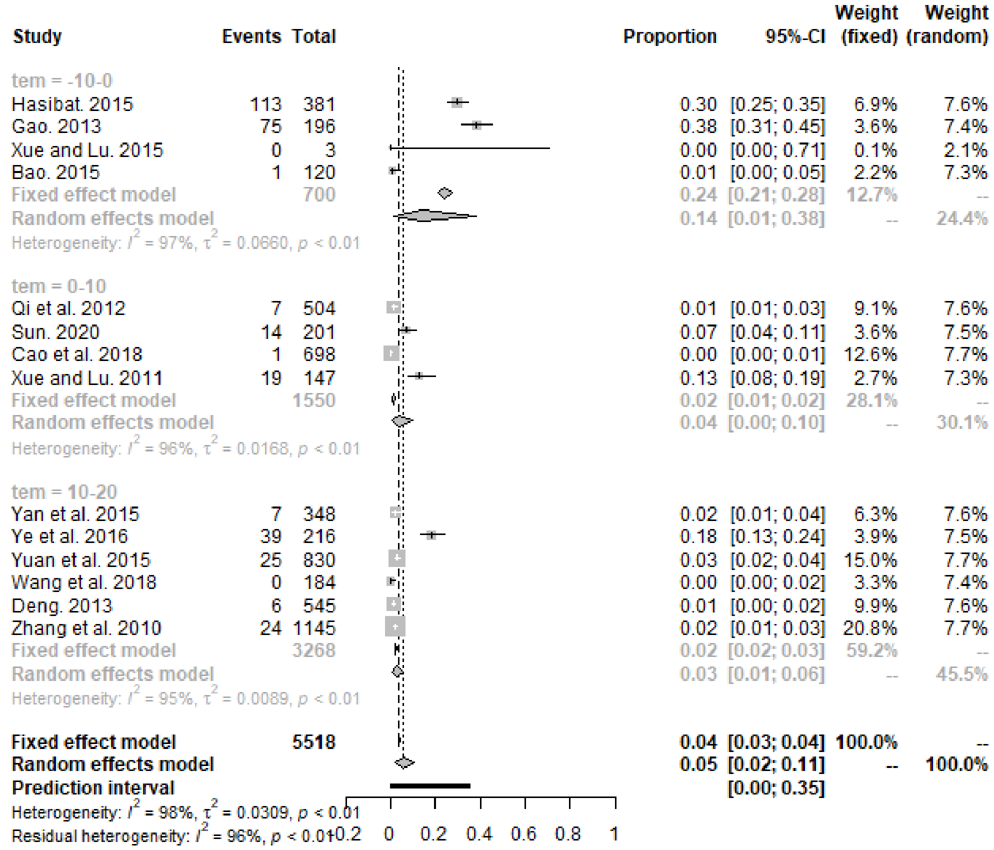

Figure S15. Forest plot of prevalence of the *Brucella* in dogs for minimum temperature

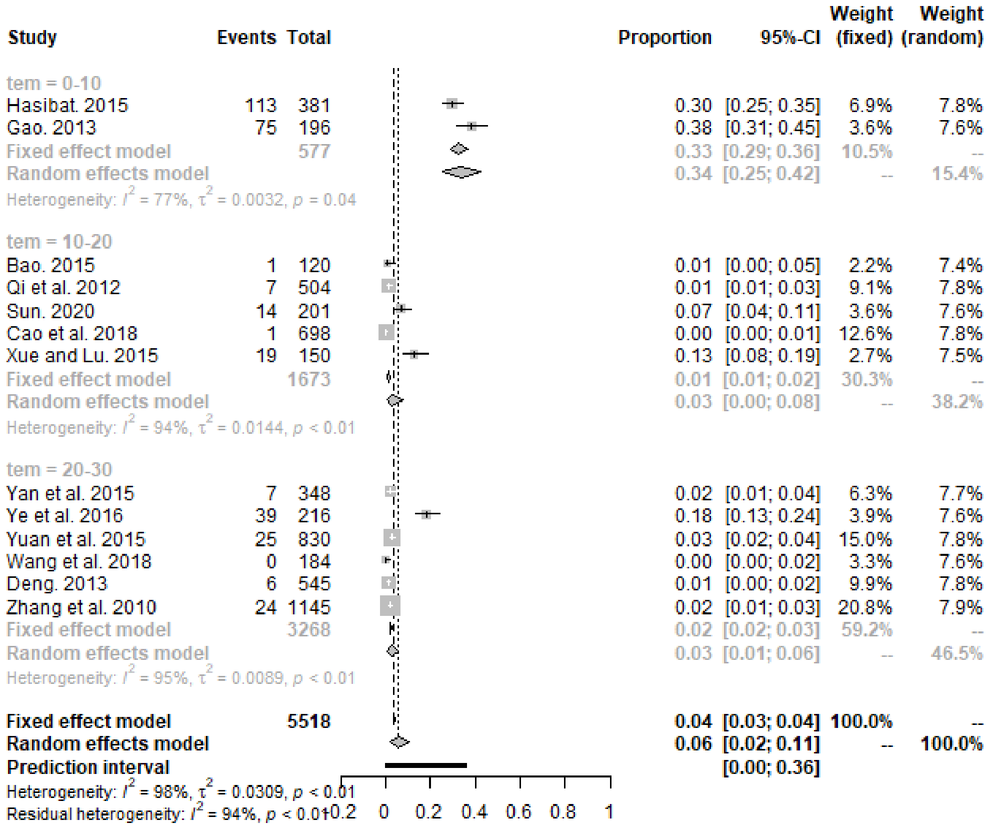

Figure S16. Forest plot of prevalence of the *Brucella* in dogs for maximum temperature
