# Supplementary material for: Prevalence of Brucella in dogs in China: a systematic review and meta-analysis—Epidemiological analysis of canine brucellosis
Source: Front Vet Sci. 2025 Feb 13;11:1515405. doi: 10.3389/fvets.2024.1515405 (PMC11866426; doi:10.3389/fvets.2024.1515405)
Supplement: Supplementary file 1 [file Data_Sheet_1.zip › Supporting information/S7. Funnel plot.pdf]

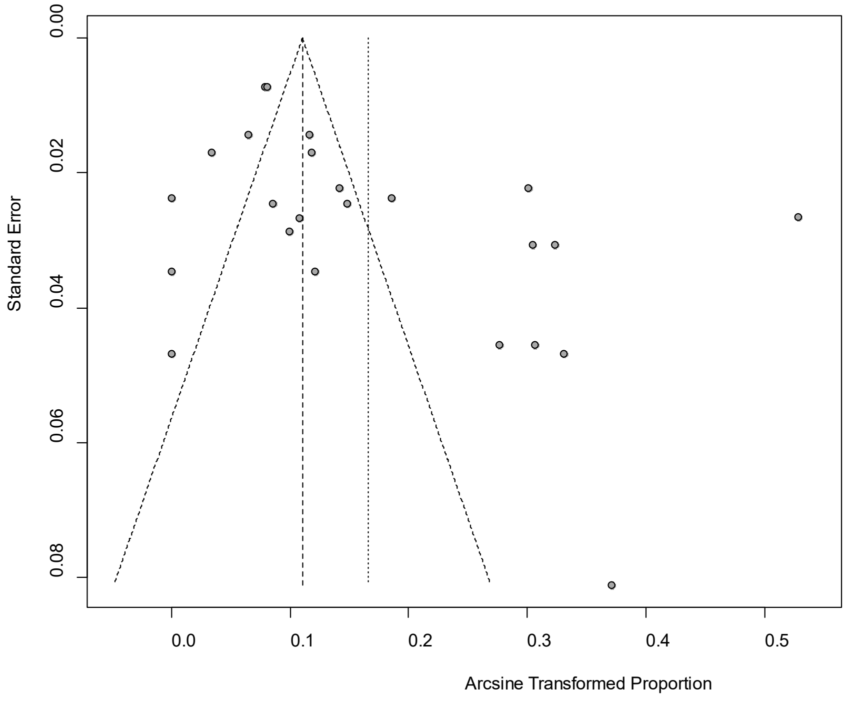

Figure S1. Funnel plot with pseudo 95% confidence limits intervals for the examination of publication bias of *Brucella* type

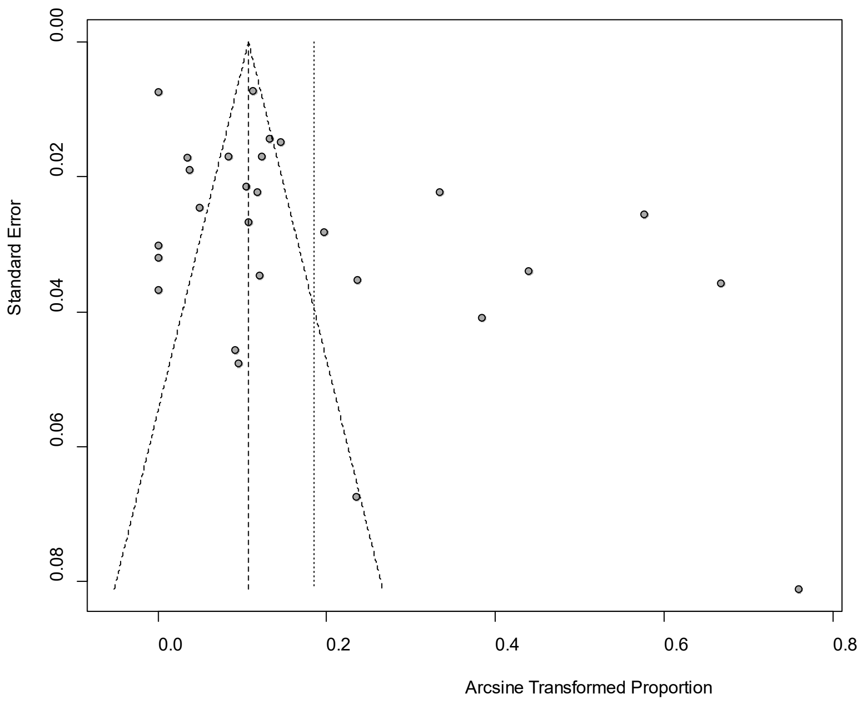

Figure S2. Funnel plot with pseudo 95% confidence limits intervals for the examination of publication bias of sampling year

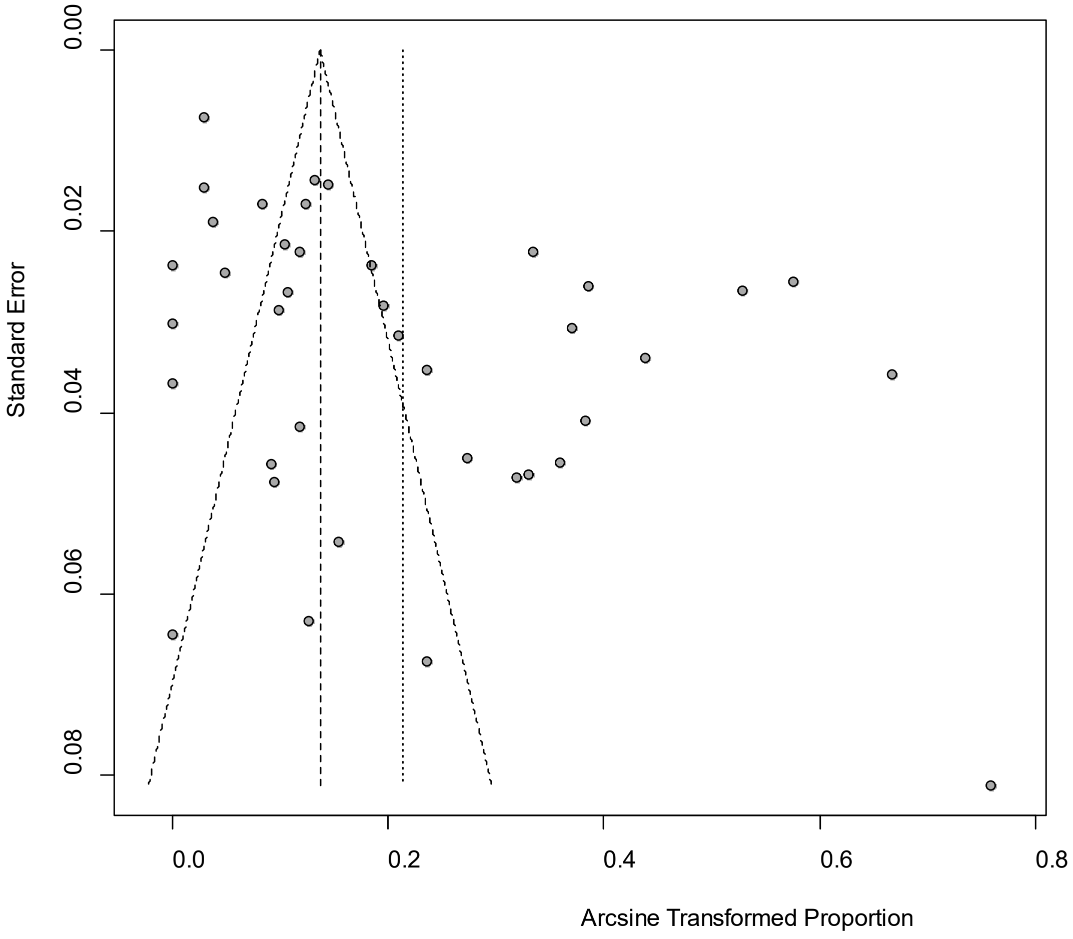

Figure S3. Funnel plot with pseudo 95% confidence limits intervals for the examination of publication bias of region

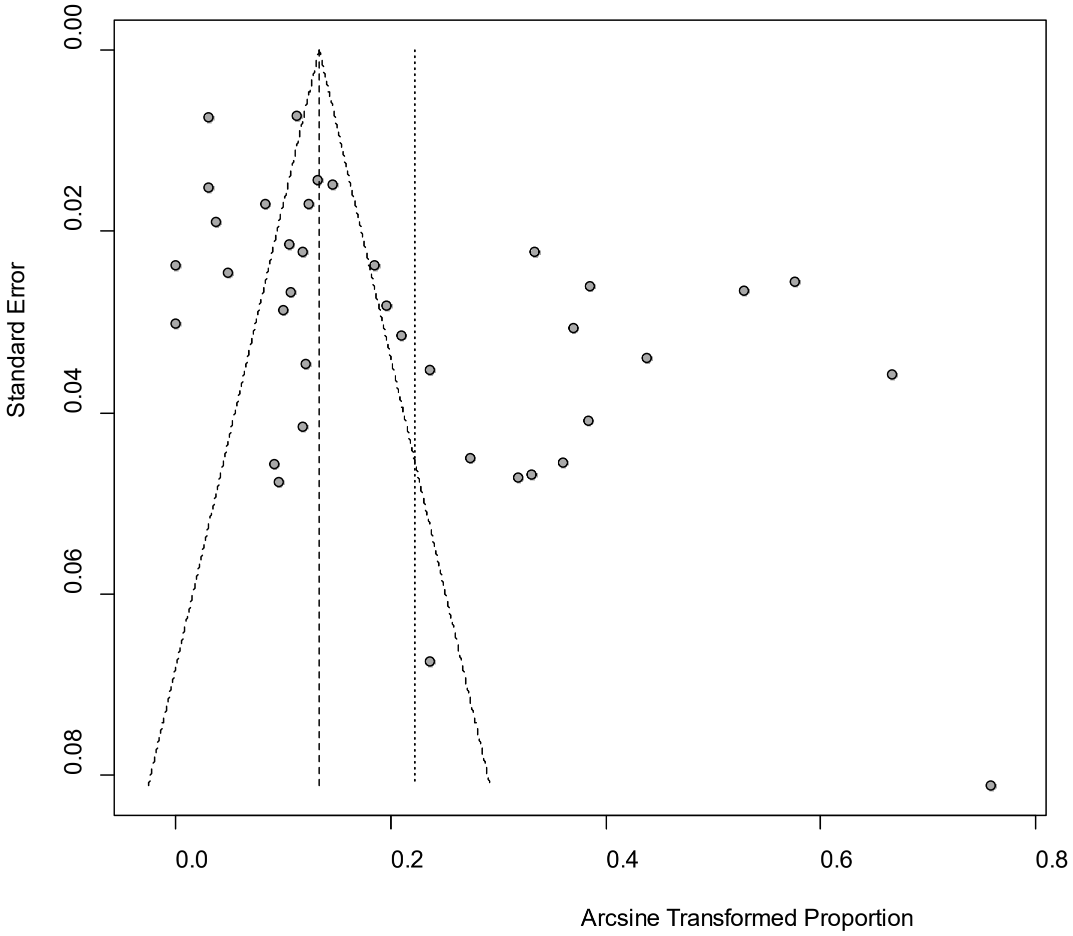

Figure S4. Funnel plot with pseudo 95% confidence limits intervals for the examination of publication bias of detection method

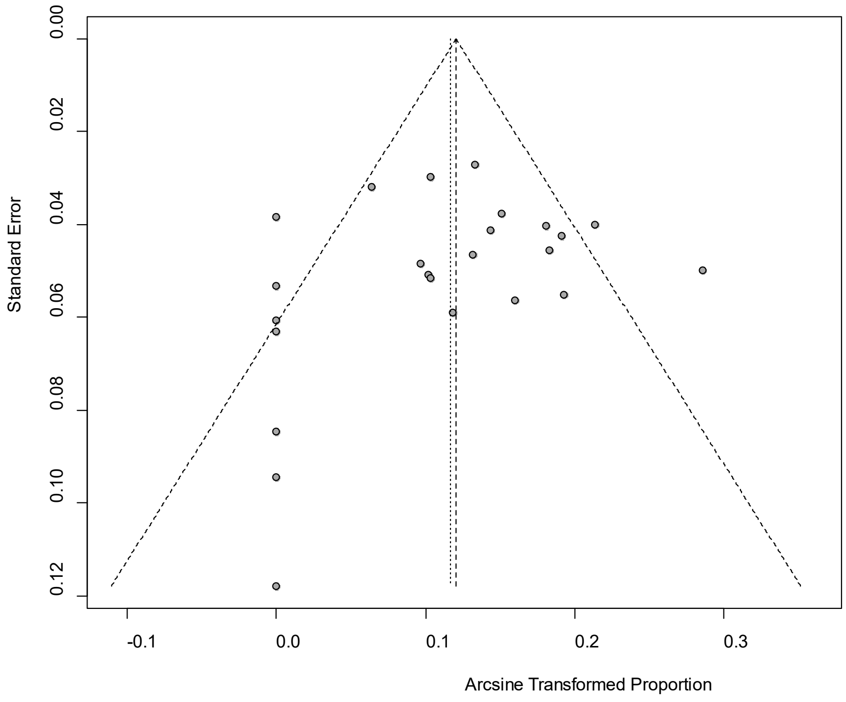

Figure S5. Funnel plot with pseudo 95% confidence limits intervals for the examination of publication bias of age

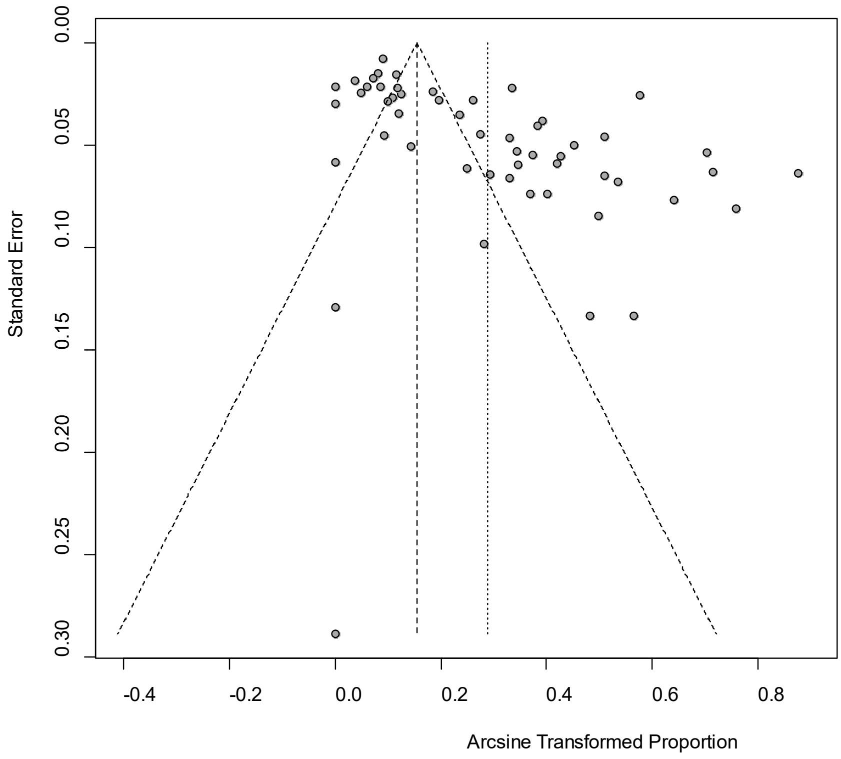

Figure S6. Funnel plot with pseudo 95% confidence limits intervals for the examination of publication bias of farming mode

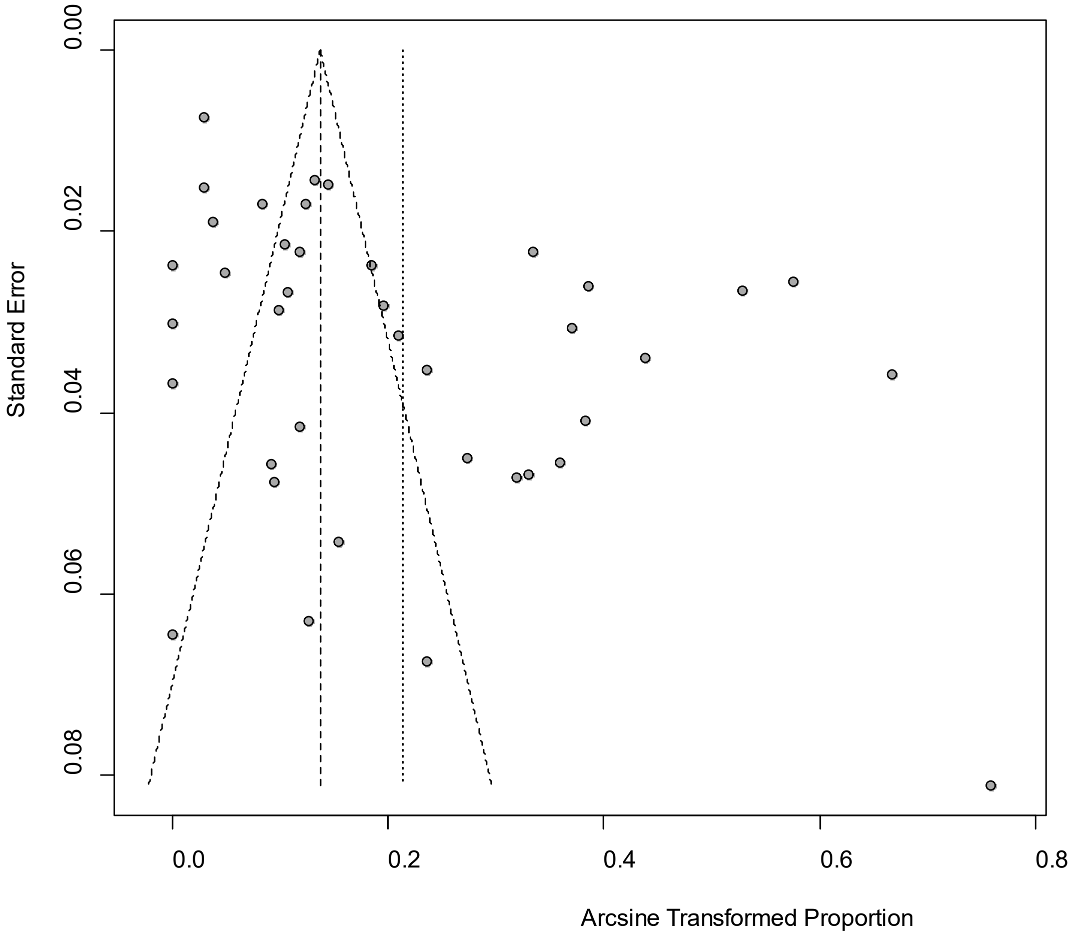

Figure S7. Funnel plot with pseudo 95% confidence limits intervals for the examination of publication bias of province

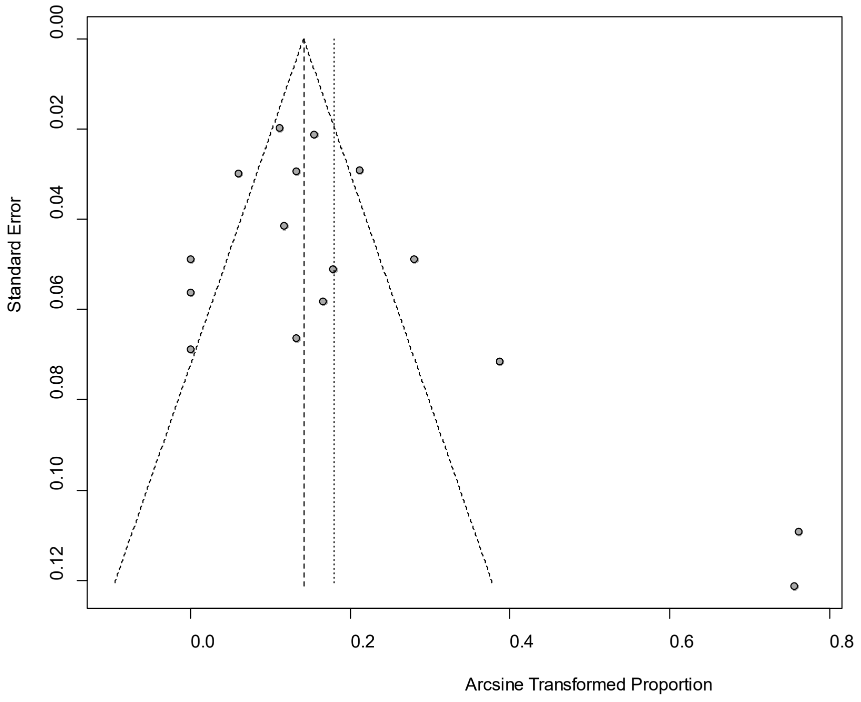

Figure S8. Funnel plot with pseudo 95% confidence limits intervals for the examination of publication bias of gender

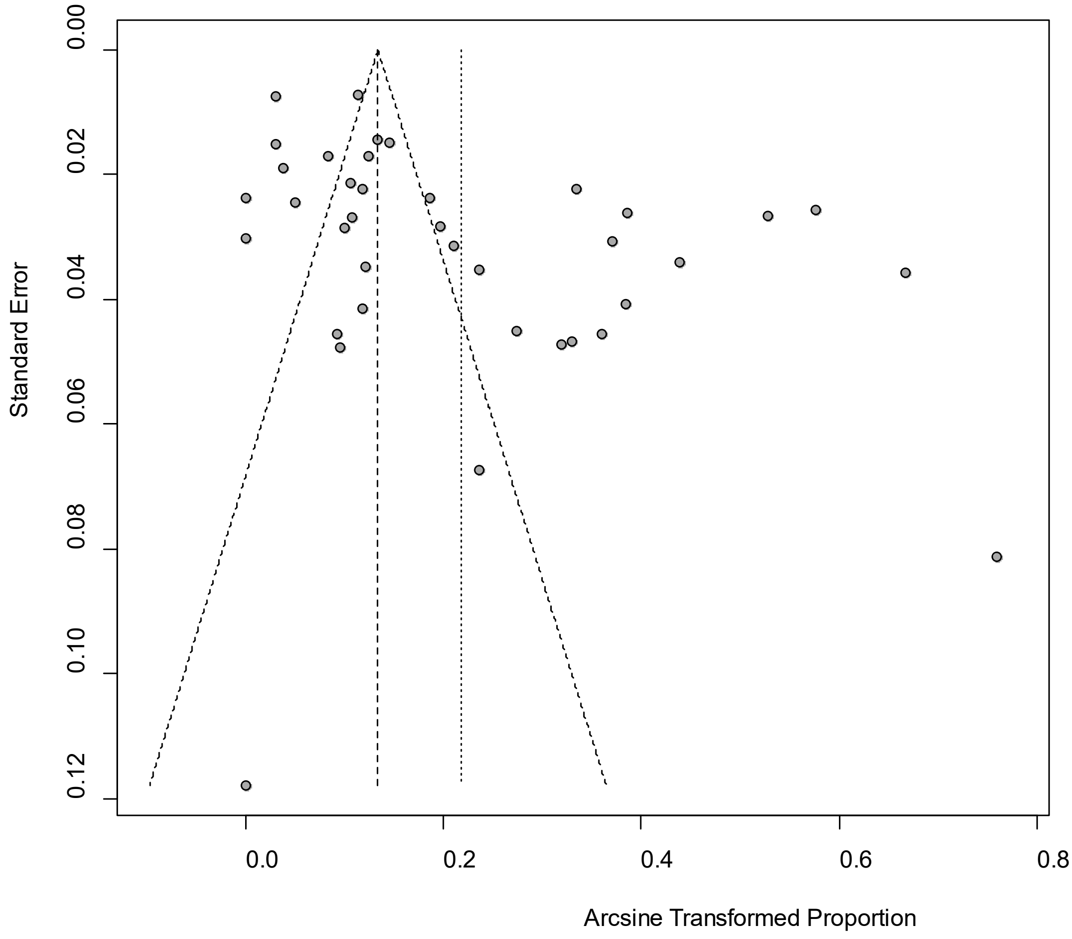

Figure S9. Funnel plot with pseudo 95% confidence limits intervals for the examination of publication bias of quality

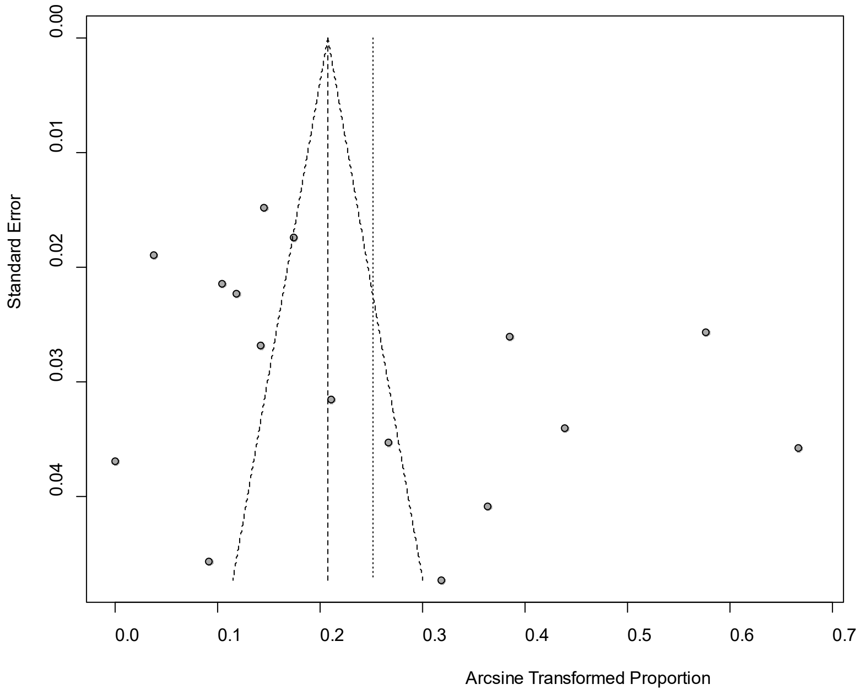

Figure S10. Funnel plot with pseudo 95% confidence limits intervals for the examination of publication bias of Northern Latitude

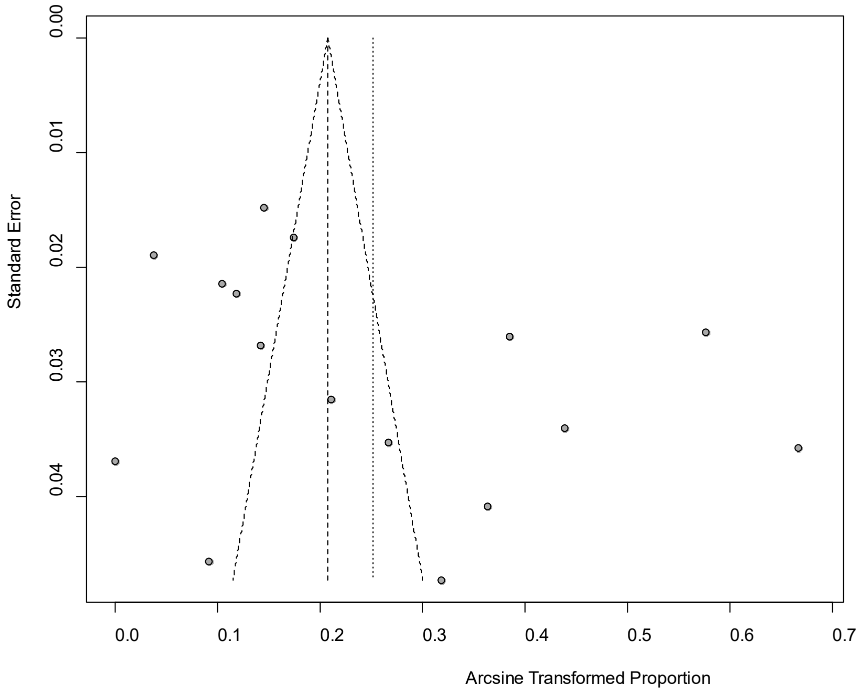

Figure S11. Funnel plot with pseudo 95% confidence limits intervals for the examination of publication bias of Eastern Longitude

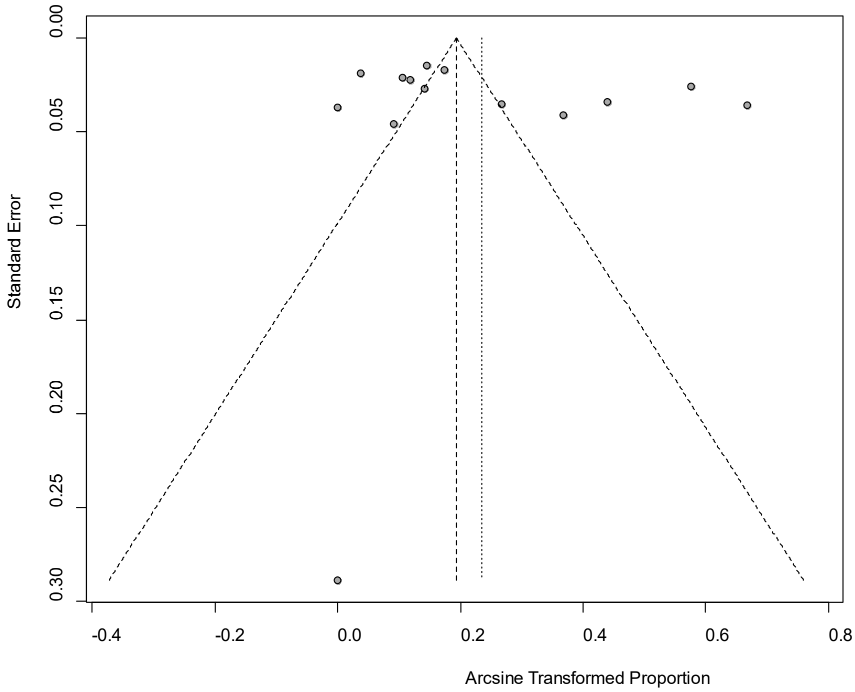

Figure S12. Funnel plot with pseudo 95% confidence limits intervals for the examination of publication bias of Rainfall

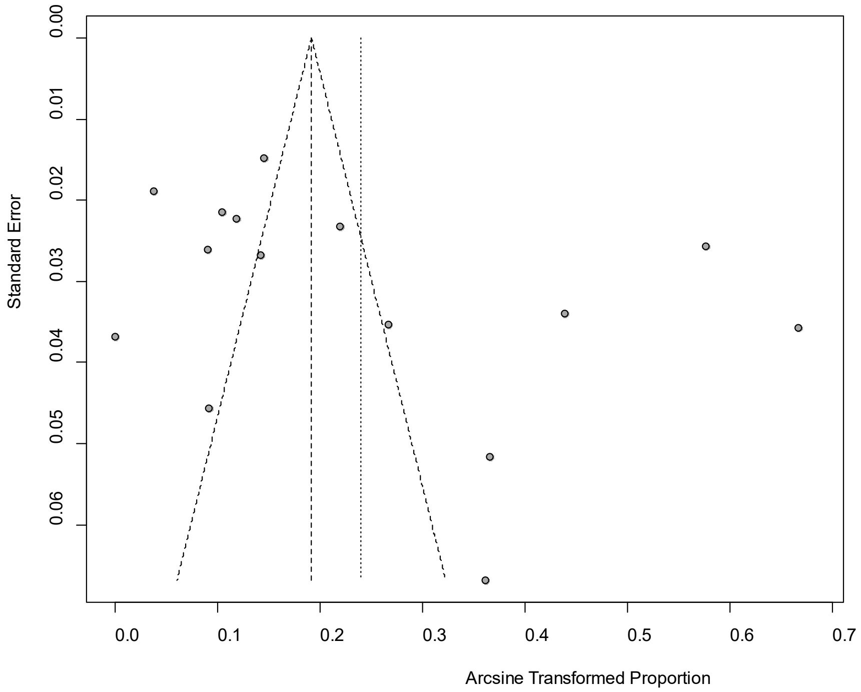

Figure S13. Funnel plot with pseudo 95% confidence limits intervals for the examination of publication bias of humidity

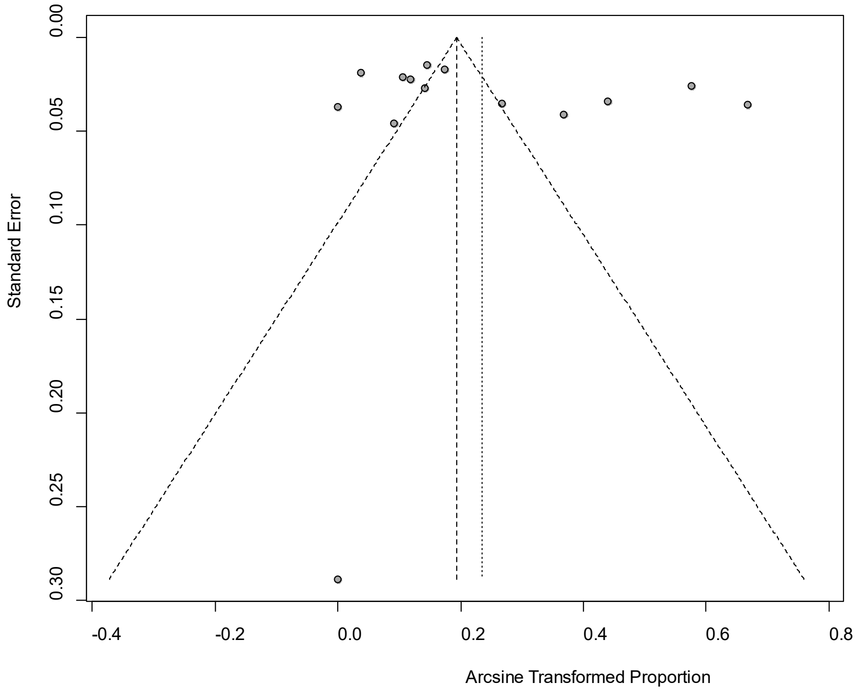

Figure S14. Funnel plot with pseudo 95% confidence limits intervals for the examination of publication bias of temperature

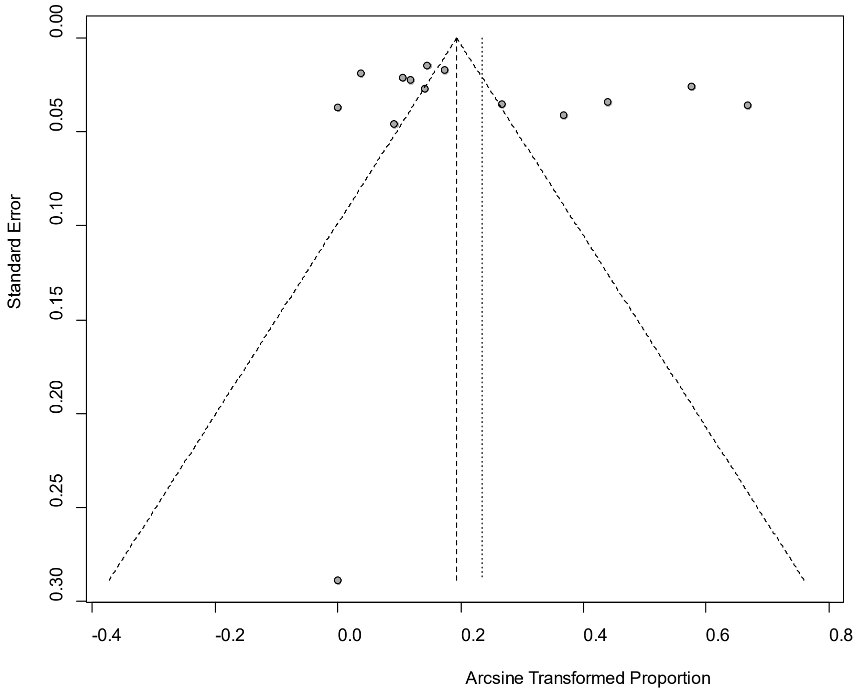

Figure S15. Funnel plot with pseudo 95% confidence limits intervals for the examination of publication bias of minimum temperature

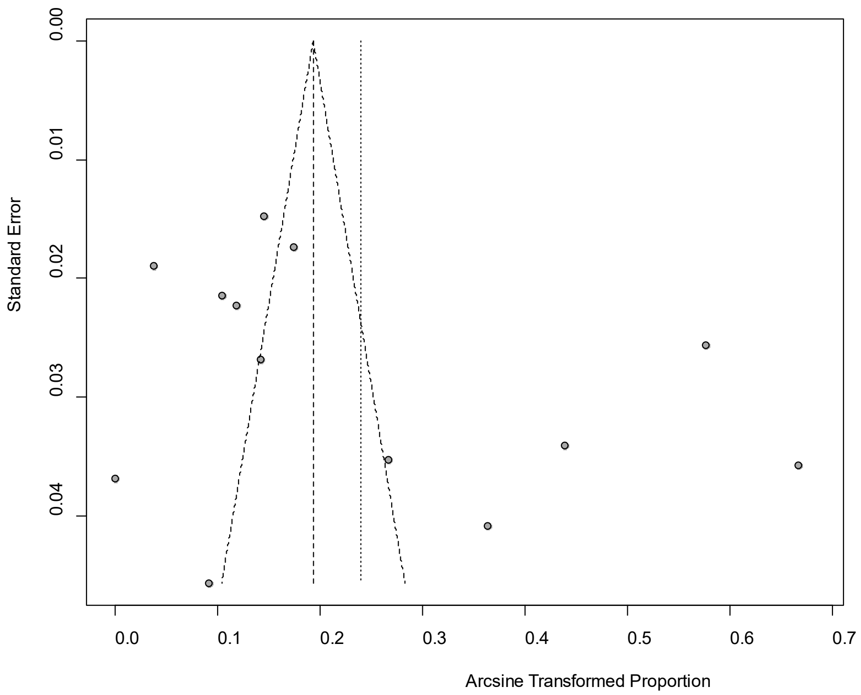

Figure S16. Funnel plot with pseudo 95% confidence limits intervals for the examination of publication bias of maximum temperature
