# Supplementary material for: Prevalence of Brucella in dogs in China: a systematic review and meta-analysis—Epidemiological analysis of canine brucellosis
Source: Front Vet Sci. 2025 Feb 13;11:1515405. doi: 10.3389/fvets.2024.1515405 (PMC11866426; doi:10.3389/fvets.2024.1515405)
Supplement: Supplementary file 1 [file Data_Sheet_1.zip › Supporting information/Table S1-S4.pdf]

Table S1. PRISMA Checklist item.

Section/topic	#	Checklist item	Reported on page #
**TITLE**			
Title	1	Prevalence of *Brucella* in dogs in China: A systematic review and meta-analysis	1
**ABSTRACT**			
Structured summary	2	Provide a structured summary including, as applicable: background; objectives; data sources; study eligibility criteria, participants, and interventions; study appraisal and synthesis methods; results; limitations; conclusions and implications of key findings; systematic review registration number.	2
**INTRODUCTION**			
Rationale	3	Describe the rationale for the review in the context of what is already known.	3-4
Objectives	4	Provide an explicit statement of questions being addressed with reference to participants, interventions, comparisons, outcomes, and study design (PICOS).	3-4
**METHODS**			
Protocol and registration	5	Indicate if a review protocol exists, if and where it can be accessed (e.g., Web address), and, if available, provide registration information including registration number.	4-7
Eligibility criteria	6	Specify study characteristics (e.g., PICOS, length of follow-up) and report characteristics (e.g., years considered, language, publication status) used as criteria for eligibility, giving rationale.	4-7
Information sources	7	Describe all information sources (e.g., databases with dates of coverage, contact with study authors to identify additional studies) in the search and date last searched.	4-7
Search	8	Present full electronic search strategy for at least one database, including any limits used, such that it could be repeated.	4-7
Study selection	9	State the process for selecting studies (i.e., screening, eligibility, included in systematic review, and, if applicable, included in the meta-analysis).	4-7

Data collection process	10	Describe method of data extraction from reports (e.g., piloted forms, independently, in duplicate) and any processes for obtaining and confirming data from investigators.	4-5
Data items	11	List and define all variables for which data were sought (e.g., PICOS, funding sources) and any assumptions and simplifications made.	4-5
Risk of bias in individual studies	12	Describe methods used for assessing risk of bias of individual studies (including specification of whether this was done at the study or outcome level), and how this information is to be used in any data synthesis.	5-7
Summary measures	13	State the principal summary measures (e.g., risk ratio, difference in means).	5-7
Synthesis of results	14	Describe the methods of handling data and combining results of studies, if done, including measures of consistency (e.g., I^2) for each meta-analysis.	5-7
Risk of bias across studies	15	Specify any assessment of risk of bias that may affect the cumulative evidence (e.g., publication bias, selective reporting within studies).	5-7
Additional analyses	16	Describe methods of additional analyses (e.g., sensitivity or subgroup analyses, meta-regression), if done, indicating which were pre-specified.	5-7
**RESULTS**			
Study selection	17	Give numbers of studies screened, assessed for eligibility, and included in the review, with reasons for exclusions at each stage, ideally with a flow diagram.	7, Fig 1
Study characteristics	18	For each study, present characteristics for which data were extracted (e.g., study size, PICOS, follow-up period) and provide the citations.	7, Table 2
Risk of bias within studies	19	Present data on risk of bias of each study and, if available, any outcome level assessment (see item 12).	7-10, Table 3
Results of individual studies	20	For all outcomes considered (benefits or harms), present, for each study: (a) simple summary data for each intervention group (b) effect estimates and confidence intervals, ideally with a forest plot.	7-10, Table 3
Synthesis of results	21	Present results of each meta-analysis done, including confidence intervals and measures of consistency.	7-10, Table 3
Risk of bias across studies	22	Present results of any assessment of risk of bias across studies (see Item 15).	9-10, Fig 5, Fig 6, and Fig 7

Additional analysis	23	Give results of additional analyses, if done (e.g., sensitivity or subgroup analyses, meta-regression [see Item 16]).	10-11, Fig 2, Fig 3, Fig 4
**DISCUSSION**			
Summary of evidence	24	Summarize the main findings including the strength of evidence for each main outcome; consider their relevance to key groups (e.g., healthcare providers, users, and policy makers).	21-22
Limitations	25	Discuss limitations at study and outcome level (e.g., risk of bias), and at review-level (e.g., incomplete retrieval of identified research, reporting bias).	10-21
Conclusions	26	Provide a general interpretation of the results in the context of other evidence, and implications for future research.	11-15
**FUNDING**			
Funding	27	Describe sources of funding for the systematic review and other support (e.g., supply of data); role of founders for the systematic review.	22-23

From: Moher D, Liberati A, Tetzlaff J, Altman DG, The PRISMA Group (2009). Preferred Reporting Items for Systematic Reviews and Meta-Analyses: The PRISMA Statement. PLoS Med 6(6): e1000097. doi:10.1371/journal.pmed1000097

For more information, visit: **www.prisma-statement.org**.

Table S2. The code in R for meta-analysis.

Logarithmic conversion (PLN)	rate<-transform [m1, log=log(event/n)];<br>shapiro.test(rate$log)
Logit transformation (PLOGIT)	rate<-transform{m1, logit=log[(event/n)/(1-event/n)]};<br>shapiro.test(rate$logit)
Arcsine transformation (PAS)	rate<-transform{m1, arcsin.size=asin[sqrt(event/(n+1))]};<br>shapiro.test(rate$arcsin)
Double-arcsine transformation (PFT)	rate<-<br>transform{m1,darcsin=0.5*[asin(sqrt(event/(n+1)))+asin((sqrt(event+1)/<br>(n+1)))]};<br>shapiro.test(rate$darcsin)
No transformation (PRAW)	rate<-transform[m1, r= event/n];<br>shapiro.test(rate$r)
Forest plots	forest [meta1, xlim=c(-0.4, 1)]
Funnel chart	funnel (meta1)

Egger's test	metabias (meta1, method="linreg")
The sensitivity analysis	metainf (meta1, pooled = "random") forest (metainf (meta1, pooled = "random"), xlim=c(0, 0.2))
Subgroup analysis	meta1<-metaprop(event, n, study, data=rate, sm="PLN", incr=0.5, allincr=TRUE, addincr=FALSE, title="", byvar= subgroup title, print.byvar=TRUE)
Meta-regression analysis	metareg (meta1, ~covariate title)

Table S3. Included studies and quality scores.

	Reference ID	No. tested	No. positive	Prevalence	Study design	Random sampling or not	the detection method clear or not	Sampled method detailedly or not	the timing of the sampling clear or not	four or more risk factors or not	Score	Study Quality
1	Li et al. (2006)	443	0	0	Cross sectional	0	1	0	0	0	1	low
2	Zhang et al. (2014)	112	11	0.098214286	Cross sectional	0	1	0	0	1	2	middle
3	Wang et al. (2018)	18	0	0	Cross sectional	0	0	0	1	1	2	middle
4	Chen et al. (2013)	267	35	0.131086142	Cross sectional	0	1	0	0	1	2	middle
5	Huang et al. (2019)	502	54	0.107569721	Cross sectional	0	1	0	0	1	2	middle
6	Liang. (2012)	442	15	0.033936652	Cross sectional	0	1	0	0	1	2	middle
7	Wang et al. (2011)	315	12	0.038095238	Cross sectional	0	1	0	1	0	2	middle

8	Hu et al. (2004)	305	3	0.009836066	Cross sectional	0	1	0	0	1	2	middle
9	Che et al. (2015)	252	11	0.043650794	Cross sectional	1	1	1	0	0	3	middle
10	Gao. (2013)	196	75	0.382653061	Cross sectional	0	1	0	1	1	3	middle
11	Liao et al. (2019)	368	52	0.141304348	Cross sectional	1	1	0	0	1	3	middle
12	Yan et al. (2015)	348	4	0.011494253	Cross sectional	0	1	0	1	1	3	middle
13	Ye et al. (2016)	216	39	0.180555556	Cross sectional	0	1	0	1	1	3	middle
14	Cao et al. (2018)	698	1	0.001432665	Cross sectional	0	1	0	1	1	3	middle
15	Deng. (2013)	545	6	0.011009174	Cross sectional	0	1	0	1	1	3	middle
16	Wang et al. (2014)	38	18	0.473684211	Cross sectional	0	1	0	1	1	3	middle
17	Xu et al. (2014)	145	2	0.013793103	Cross sectional	1	1	0	0	1	3	middle
18	Yang et al. (2015)	110	1	0.009090909	Cross sectional	0	1	0	1	1	3	middle
19	Zhang et al. (2016)	860	13	0.015116279	Cross sectional	0	1	0	1	1	3	middle
20	Liu et al. (2017)	121	15	0.123966942	Cross sectional	1	1	0	0	1	3	middle
21	Tao et al. (2017)	114	12	0.105263158	Cross sectional	1	1	0	0	1	3	middle
22	Wang et al. (2018)	354	90	0.254237288	Cross sectional	1	1	0	0	1	3	middle
23	Wang. (2013)	55	3	0.054545455	Cross sectional	0	1	0	1	1	3	middle
24	Lu et al. (2014)	1094	1	0.000914077	Cross sectional	0	1	0	1	1	3	middle
25	Chen et al. (2018)	4508	4	0.000887311	Cross sectional	0	1	0	1	1	3	middle
26	Di et al. (2011)	4750	60	0.012631579	Cross sectional	0	1	0	1	1	3	middle
27	Zhang et al. (2010)	1145	24	0.020960699	Cross sectional	0	1	0	1	1	3	middle
28	Xiang et al. (2009)	1200	21	0.0175	Cross sectional	0	1	0	1	1	3	middle
29	He et al. (2009)	415	1	0.002409639	Cross sectional	0	1	0	1	1	3	middle
30	Liu et al. (2002)	123	9	0.073170732	Cross sectional	0	1	0	1	1	3	middle
31	Bao. (2015)	120	1	0.008333333	Cross sectional	1	1	0	1	1	4	high
32	Hasibat. (2015)	381	113	0.296587927	Cross sectional	1	1	0	1	1	4	high
33	Qi et al. (2012)	504	7	0.013888889	Cross sectional	1	1	0	1	1	4	high
34	Sun. (2020)	201	11	0.054726368	Cross sectional	0	1	1	1	1	4	high
35	Yuan et al. (2015)	870	6	0.006896552	Cross sectional	0	1	1	1	1	4	high

36	Wang et al. (2017)	275	0	0	Cross sectional	0	1	1	1	1	4	high
37	Xue and Lu (2011)	150	21	0.14	Cross sectional	1	1	0	1	1	4	high
38	Xu et al. (2020)	208	3	0.014423077	Cross sectional	1	1	1	1	1	5	high

Y*: Yes; N*: No.

References

1. Li K H, Xue X, Ju G N, et al. Serological survey of major canine epidemics in the Shanghai area. Shanghai Journal of Animal Husbandry and Veterinary Medicine, 2006, (05):43-44. (in China)
2. Zhang J, Xiao X, Cai Q B, et al. Comparison of immunogold standard test paper test and PCR test for the investigation of canine brucellosis infection status in Kunming area. Heilongjiang Animal Science And veterinary Medicine,2014, (07):105-107+210.DOI: 10.13881/j.cnki.hljxmsy.2014.0106. (in China)
3. Lu X H, Deng G Q, Wang C J, et al. Surveillance and analysis of major zoonotic diseases of urban companion animals. Heilongjiang Animal Science and Veterinary Medicine. 2018(10):224-225.
4. Chen Y, Lin Z L, Deng M Q, et al. Serological survey of Brucella in experimental Beagles. Dog Breeding, 2013(4):6-8. (in China)
5. Hang D H, Guo H, Zhang W, et al. Seroepidemiological Investigation on Brucellosis in Canine in Jinan City of Shandong Province from 2016 to 2017. China Animal Health Inspection,2019,36(07):15-19. (in China)
6. Liang Y. Serological investigation of influenza, chlamydia and brucellosis in pet dogs in Shenzhen, China. South China Agricultural University, 2012.DOI:10.7666/d. Y2245473. (in China)
7. Wang Y, Chen G C, Liu Z B, et al. Survey on the prevalence of brucellosis in some large hydropower project areas in Guizhou Province. Journal of Medical Pest Control | J Med Pest Contrl,2011,27(09):850. (in China)
8. Hu Z Y, Li Y J, Ma X R, et al. Serological survey of brucellosis in sheepdog breeds in a Himalayan dry otter plague infected area. Health Vocational Education,

2004, (23):90. (in China)

9. Che X J, Guo H L, Meng L M, et al. Gansu Tianshui canine brucellosis and three other zoonotic diseases serological survey. Chinese Journal of Veterinary Medicine,2015,51(01):81. (in China)
10. Gao M H. Serologic Investigation of Brucelosis Infection in Canine in Hulun Buir. Chinese Journal of Veterinary Medicine,2013,49(05):59-61. (in China)
11. Liao S T, Xia L N, Xu J G, et al. Epidemiological survey of canine brucellosis in Wuchang area, Xinjiang, China. Hubei Journal of Animal and Veterinary Sciences,2019,40(01): 10-11.DOI: 10.16733/j.cnki.issn1007-273x.2019.01.003. (in China)
12. Yan W, Zhao S S, Liu J, et al. Prevalence of important zoonotic bacterial diseases in pets in Taizhou, Jiangsu Province, China. Jiangsu Agricultural Sciences,2015,43(03): 198-200.DOI: 10.15889/j.issn.1002-1302.2015.03.064. (in China)
13. Ye F, Ma X J, Wang J, et al. Serological survey of canine brucellosis in the area around Urumqi, Xinjiang, China. Chinese Journal of Veterinary Medicine,2016,52(12):80-81. (in China)
14. Cao X A, Li S, Li Z C, et al. Enzootic situation and molecular epidemiology of Brucella in livestock from 2011 to 2015 in Qingyang, China. Emerging microbes & infections,2018,7(1):58. (in China)
15. Deng G J. Serological survey on canine brucellosis in Tai Po County. Contemporary Animal Husbandry, 2013, (23):79. (in China)
16. Wang N, Han J S, Lv Y L, et al. The isolation of Brucella from pet dogs in Beijing. Chinese Journal of Preventive Veterinary Medicine,2014,36(06):490-492. (in China)
17. Xu G J, Su Y L. Serological survey of brucellosis infection in dogs in the Ximen area. Modern Animal Husbandry, 2014, (08): 59.DOI: 10.14070/j.cnki.15-1150.2014.08.133. (in China)
18. Yang Y, Xu X Y, Yu S, et al. Serological survey of canine brucellosis in some areas of Sichuan and diagnosis of typical cases. Animal Husbandry and Veterinary Medicine,2015,47(05):99-101. (in China)

19. Zhang Q Y, Ni X Q, Zeng D, et al. Serosurvey of canine brucellosis in Chengdu and development of real-time PCR for rapid detection of Brucella. Veterinary Science in China,2016,46(04): 430-435.DOI: 10.16656/j.issn.1673-4696.2016.04.005. (in China)
20. Liu L Y, Lu G L, Wang J, et al. Investigation on the prevalence of Brucella, Salmonella and Toxoplasma gondii infection in dogs in Urumqi area. Animal Husbandry & Veterinary Medicine,2017,49(09):98-100. (in China)
21. Tao J, Yin F Z, Huang Z, et al. Serological survey of brucellosis in domesticated dogs in an area of Shanghai, China. Shanghai Journal of Animal Husbandry and Veterinary Medicine,2017, (05): 68-70.DOI: 10.14170/j.cnki.cn31-1278/s.2017.05.023. (in China)
22. Wang T, Zhang Y S, Wang L H, et al. Epidemiological Investigation of Canine Brucellosis in Urumqi City of Xinjiang. China Animal Health Inspection，2018，35（6）：8-11. (in China)
23. Wang Y. Study on the serological surveillance and molecular characteristics of Brucella in Guizhou Province. Guizhou Medical University. (in China)
24. Lu W Y, He F Y, Li S E. Epidemiological survey of brucellosis in Qingyang City between 2009 and 2013. Journal of Animal Science and Veterinary Medicine,2014,33(03):73-74+76. (in China)
25. Chen S W, Zhou Z H, Liu X Q, et al. Survey on serum antibodies against brucellosis in animals in Dongguan City, China. Chinese Journal of Animal Husbandry and Veterinary Medicine, 2018, (08):12-14. (in China)
26. Di D D, Fan W X, Cui B Y, et al. Epidemiological survey of canine Borrelia burgdorferi infection in some areas of China． Animal Husbandry and Veterinary Medicine，2011，43 (5): 83 － 85. (in China)
27. Zhang X P, Cai Y Q, Zhu Y Q, et al. Serological survey of brucellosis in dogs and cats in Dongguan city. Heilongjiang Animal Science and Veterinary Medicine, 2010, (16): 72-73.DOI: 10.13881/j.cnki.hljxmsy.2010.16.026. (in China)
28. Xiang F, Wu Q M, Wang Z, et al. Results of serological survey and analysis of canine brucellosis in the Beijing area,2009,19(06):34-38. (in China)

29. He D, Wei H T, Zhao J Y, et al. Serological survey of canine brucellosis in Beijing area. Chinese Journal of Veterinary Medicine, 2009,45(02):64-65. (in China)
30. Liu P, Yang J T, Yang L. Clinical investigation of Brucella canis infections in outpatient dogs in the Shanghai area. Chinese Journal of Veterinary Medicine,2002,(11):41-42. (in China)
31. Bao S T. Investigation of brucellosis in sheepdogs in Men yuan County, Qinghai Province, China. Animals Breeding and Feed,2015, (10): 71-72.DOI: 10.13300/j.cnki.cn42-1648/s.2015.10.032. (in China)
32. Hasibat. Epidemiological investigation of brucellosis in Turko shepherd dogs in pastoral areas of Hejing County. Today Animal Husbandry and Veterinary Medicine, 2016, (03):63. (in China)
33. Qi h x, Zhang H Y, Deng X Y, et al. Dog and Cat Brucellosis Epidemiology. Beijing Agriculture,2012, (18):98-99. (in China)
34. Sun C Y. Investigation on Brucella Infection of Dogs And Cats in Shenyang of Liaoning Province. Shenyang Agricultural University, 2020.DOI: 10.27327/d.cnki.gshnu.2020.000587. (in China)
35. Yuan T H Z. Serology Survey of Canine Toxoplasmosis and Brucellosis in Hunan Province. Hunan Agricultural University,2015. (in China)
36. Wang Y Q, Zhang H Y, Tang Y R, et al. Epidemiological Survey of Zoonoses in Animal Treatment Facilities in Chaoyang District, 2016. Chinese Abstracts of Animal Husbandry and Veterinary Medicine,2017,33(10):12-13+21. (in China)
37. Xue Y P, Wang P, Lu W Y. Survey on the Current Status of Canine Brucellosis in Some Cities of Gansu Province and Reflections. Journal of Animal Science and Veterinary Medicine,2011,30(01):64-65. (in China)
38. Xu T, Mao A G, Wang X, et al. Investigation of serum antibodies to brucellosis in police dogs in Yunnan, Guizhou and Sichuan provinces. Chinese Journal of Veterinary Medicine,2020,56(08):43-44. (in China)

Table S4. Egger's for publication bias.

slope	bias	se. bias	t	df	*P*-value
0.027	6.24	1.68	3.72	36	0.000686
